# Functional and modular analyses of diverse endoglucanases from *Ruminococcus albus* 8, a specialist plant cell wall degrading bacterium

**DOI:** 10.1038/srep29979

**Published:** 2016-07-21

**Authors:** Michael Iakiviak, Saravanan Devendran, Anna Skorupski, Young Hwan Moon, Roderick I. Mackie, Isaac Cann

**Affiliations:** 1 Energy Biosciences Institute, University of Illinois at Urbana-Champaign, Urbana, Illinois 61801, USA; 2 Department of Animal Sciences, University of Illinois at Urbana-Champaign, Urbana, Illinois 61801, USA; 3 Carl R. Woese Institute for Genomic Biology, University of Illinois at Urbana-Champaign, Urbana, Illinois 61801, USA; 4 School of Molecular and Cellular Biology, University of Illinois at Urbana-Champaign, Urbana, Illinois 61801, USA; 5 Department of Microbiology, University of Illinois at Urbana-Champaign, Urbana, Illinois 61801, USA

## Abstract

*Ruminococcus albus* 8 is a specialist plant cell wall degrading ruminal bacterium capable of utilizing hemicellulose and cellulose. Cellulose degradation requires a suite of enzymes including endoglucanases, exoglucanases, and β-glucosidases. The enzymes employed by *R. albus* 8 in degrading cellulose are yet to be completely elucidated. Through bioinformatic analysis of a draft genome sequence of *R. albus* 8, seventeen putatively cellulolytic genes were identified. The genes were heterologously expressed in *E. coli*, and purified to near homogeneity. On biochemical analysis with cellulosic substrates, seven of the gene products (Ra0185, Ra0259, Ra0325, Ra0903, Ra1831, Ra2461, and Ra2535) were identified as endoglucanases, releasing predominantly cellobiose and cellotriose. Each of the *R. albus* 8 endoglucanases, except for Ra0259 and Ra0325, bound to the model crystalline cellulose Avicel, confirming functional carbohydrate binding modules (CBMs). The polypeptides for Ra1831 and Ra2535 were found to contain distantly related homologs of CBM65. Mutational analysis of residues within the CBM65 of Ra1831 identified key residues required for binding. Phylogenetic analysis of the endoglucanases revealed three distinct subfamilies of glycoside hydrolase family 5 (GH5). Our results demonstrate that this fibrolytic bacterium uses diverse GH5 catalytic domains appended with different CBMs, including novel forms of CBM65, to degrade cellulose.

Cellulose is the main structural component of the plant cell wall and represents a large source of renewable carbon^1^. As a structural polysaccharide, cellulose is recalcitrant to degradation due to the high degree of crystallinity formed by extensive hydrogen bonding, as well as the stability of the β-1,4 glycosidic linkage. During synthesis, the hydrogen-bonding network may be disrupted, forming amorphous regions that are more accessible to enzymatic attack^2^.

The degradation of cellulose mainly requires the concerted action of three classes of enzymes, namely endoglucanases, exoglucanases, and β-glucosidases, which may also harbor cellodextrinase activity. Endoglucanases (EC 3.2.1.4) randomly cleave β-1,4 linkages of cellulose to yield shorter chain products. Exoglucanases (EC 3.2.1.91) bind to the glucan chain ends and cleave off cellobiose, the repeating unit of cellulose. Thus, exoglucanases release cellobiose as the main end products. The cellobiose and cello-oligosaccharides released by the endoglucanase and the exoglucanase are further hydrolyzed into glucose monomers by β-glucosidases (EC 3.2.1.21) or cellodextrinases (EC 3.2.1.74). Though rare, processive endoglucanases are able to cleave the linkages in glucan chains in a random manner, as described for the commonly known endoglucanases, and then continue to processively release cellobiose, as described for exoglucanases^3^,^4^. Synergy in degradation of cellulose to soluble sugars or reducing ends can be detected in reactions containing β-glucosidases, endoglucanases, and exoglucanases^5^,^6^.

Cellulolytic organisms commonly possess multiple enzymes within each category of the enzymes described above^7^. Furthermore, multiple glycoside hydrolase (GH) catalytic modules can exist in a single polypeptide chain with other non-catalytic accessory modules, such as carbohydrate-binding modules (CBM). The modular arrangement of the polypeptide may improve the capacity of an enzyme to hydrolyze its substrate by increasing affinity and proximity to the substrate^8^. Carbohydrate-binding modules can exhibit specific or non-specific binding to substrates^8^,^9^, and due to their unique characteristics, CBMs have been adapted to technologies such as affinity tags and analysis of carbohydrate content of a substrate^10^. Recent reports suggest a greater diversity of CBMs than is currently known, and this is evidenced by the identification and biochemical characterization of a new CBM family designated CBM65^11^.

Ruminal microbes have evolved to degrade plant cell wall polysaccharides. Genome sequences from the major fibrolytic ruminal bacteria, *Fibrobacter succinogenes* S85, *Prevotella bryantii* B_1_4, *Prevotella ruminicola* 23, and *Ruminococcus albus* (strains 7 and 8) reveal multiple putative carbohydrate active enzymes implicated in the degradation of cellulose and hemicelluloses. Furthermore, analyses of rumen derived metagenomic sequences have led to the discovery of unique enzymes, proving the rumen as a rich source of fibrolytic enzymes^12^.

*Ruminococcus albus* is a dominant cellulolytic bacterium in the rumen, and it is able to degrade and ferment both cellulose and hemicellulose^13^. During growth of *R. albus* 7 on cellulose in continuous culture, soluble products are rapidly utilized resulting in trace amount of soluble sugars in the culture medium, suggesting depolymerization of cellulose as the rate-limiting step^14^. Adhesion of *R. albus* cells to cellulose is important for degradation and utilization of the substrate, as adhesion defective mutants cannot effectively utilize cellulose^15^,^16^. Previous work on an *R. albus* 8 adhesion defective mutant identified two proteins, Cel9B and Cel48A, as the potential cause for decreased cellulose hydrolysis^16^. These two proteins contain a novel CBM found only in *R. albus*. The unique CBM, which binds to various polysaccharides, as well as the bacterial cell wall, has been designated CBM37. The *R. albus* CBM37 is proposed to function by shuttling the catalytic domain, to which it is attached, between the bacterial cell wall and the polysaccharide substrate^17^.

Although several enzymes from related strains have been cloned and partially characterized (Supplementary Table S1), no cellulolytic enzymes from *R. albus* 8 have been analyzed in detail. In the present report, seven endoglucanases from *R. albus* 8 were biochemically characterized to determine their contribution to hydrolysis of cellulose. Furthermore, examination of the binding capacity of the endoglucanases reflected the predicted functionality of their CBMs, and truncational analysis revealed a potentially novel CBM present in Ra1831, one of the endoglucanases. Mutational analysis of the Ra1831 CBM uncovered the residues involved in substrate recognition and binding. The results presented here provide a deeper understanding of the enzymes employed by one of the major plant cell wall degrading organisms in the rumen, and the insights gained should also be of utility to efforts aimed at plant cell wall depolymerization for conversion to value added products, including biofuels.

## Results

### Domain analysis of endoglucanases from *R. albus* 8

In order to identify cellulose degrading enzymes in *R. albus* 8, we analyzed the genome of the bacterium by bioinformatics and biochemical approaches. A search through the draft genome sequence of this bacterium yielded 17 genes predicted to code for enzymes involved in cellulose hydrolysis. The genes were expressed and their products were subjected to an initial enzymatic screen with cellulosic substrates. Seven polypeptides exhibited endoglucanase activities and were selected for further analysis.

**Figure 1:**
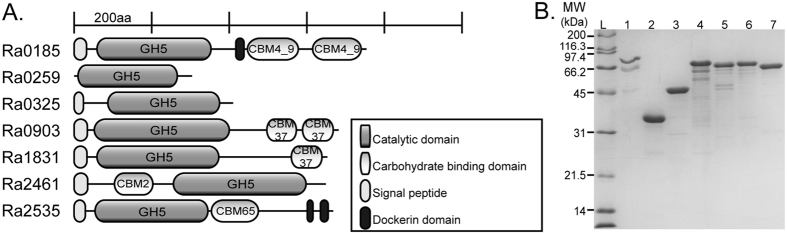
Domain architectures and purification of 7 glycoside hydrolase family 5 enzymes from *Ruminococcus albus* 8. (**A**) Domain architecture of 7 putative glycoside hydrolases predicted to be involved in cellulose hydrolysis. Domain boundaries were predicted using Pfam (http://pfam.sanger.ac.uk). Regions in light grey are signal peptides which were predicted using SignalP (http://www.cbs.dtu.dk/services/SignalP). Regions in dark grey display homology to dockerin sequences. Abbreviations: CBM, carbohydrate binding module; GH, glycoside hydrolase; Ig, immunoglobulin. (**B**) SDS-PAGE analysis of 7 putative endoglucanases purified using cobalt affinity chromatography and gel filtration. The *R. albus* 8 Ra0185, Ra0903, Ra1831, Ra2461 and Ra2535 were further purified using anion exchange chromatography. Lane L, molecular mass markers; Lane 1, Ra0185; Lane 2, Ra0259; Lane 3, Ra0325; Lane 4, Ra0903; Lane 5, Ra1831; Lane 6, Ra2461; Lane 7, Ra2535.

In Fig. 1A, the domain architecture of the 7 polypeptides demonstrated as endoglucanases (Ra0185, Ra0259, Ra0325, Ra0903, Ra1831, Ra2461, and Ra2535) in this study are presented. All of the endoglucanases possess GH5 catalytic domains; however, the presence of accessory modules and their organization varied among the polypeptides as depicted in Fig. 1A. Two endoglucanases lacked any accessory domains, Ra0259 and Ra0325. The *R. albus* 8 Ra0903 and Ra1831 both possess GH5 domains at the N-terminus and CBM37 domains at the C-terminus. The Ra0185 and Ra2535 enzymes possess dockerin-like sequences as well as CBMs C-terminal to the GH5 domain. Finally, Ra2461, contains a CBM2 N-terminal to the GH5 domain.

All the genes were cloned without the signal peptide to facilitate accumulation of the gene products in the cytoplasm of the *E. coli* strain used for protein production. Furthermore, a hexa-histidine tag (His-tag) in the plasmid was fused to the N-terminus of the recombinant protein to facilitate purification of each protein by affinity chromatography. The proteins were heterologously expressed in *E. coli* BL-21 CodonPlus RIPL and were purified to near homogeneity using immobilized metal affinity chromatography (IMAC), followed by size exclusion chromatography. Further purification was required for Ra0185, Ra0903, Ra1831, Ra2461, and Ra2535, and this was accomplished through anion exchange chromatography (Fig. 1B). For two of the endoglucanases, Ra0185 and Ra0903, some lower molecular weight bands were always present after purification, likely corresponding to degradation products of the respective enzymes.

### Quantification of PASC hydrolysis by the *R. albus* 8 enzymes

Each predicted endoglucanase was screened for hydrolysis of cellulosic substrates through overnight incubations on two different substrates, soluble carboxymethyl cellulose (CMC) and the mostly amorphous cellulosic substrate phosphoric acid swollen cellulose (PASC). Each of the enzymes depicted in Fig. 1A was capable of releasing products from the cellulosic substrates, and the end products of hydrolysis were separated and detected by TLC (Supplementary Fig. S1).

**Figure 2:**
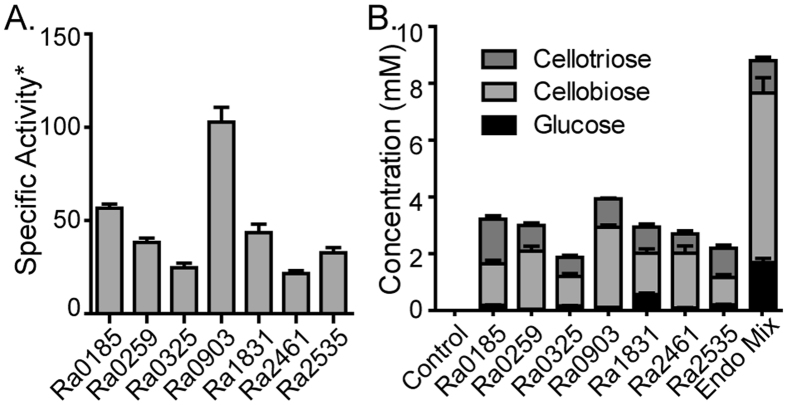
Comparison of cellulose hydrolysis by the endoglucanases of *R. albus* 8. (**A**) Specific activity (mmole of cellobiose equivalent released per minute per mmole of enzyme) of endoglucanases on phosphoric acid swollen cellulose (PASC) determined using the *para*-hydroxybenzahydrazide (PAHBAH) reducing sugar assay. (**B**) Hydrolysis of PASC (0.5% w/v) by individual endoglucanases and their mixture (0.5 μM each) was conducted for 16 hours at 37 °C. Samples were analyzed by separating the soluble products via HPAEC-PAD. Products were identified by comparison of the retention time to those of glucose, cellobiose, and cellotriose. The concentrations of products were determined by comparison of the peak area to a standard curve created using known concentrations of oligosaccharide standards.

Due to the higher release of end-products from PASC, the amorphous substrate was used to characterize the hydrolysis patterns of each endoglucanase (Fig. 2A). The endoglucanases Ra0185, Ra0259, Ra0325, Ra1831, and Ra2535 released reducing sugars at relatively similar rates, i.e., between 24.7 and 56.6 mmol of cellobiose equivalent per mmol of enzyme per minute. The enzyme with the highest activity, Ra0903 was able to release reducing sugars at 102.8 mmol cellobiose equivalents per mmol enzyme per min, while the enzyme with the lowest activity, Ra2461, released cellobiose equivalents at a rate of 21.6 mmol per mmol enzyme per min.

Endoglucanases are expected to release a mixture of longer chain oligosaccharides, ranging from cellotriose to cellohexaose^18^. Interestingly, all of the endoglucanases from *R. albus* 8 were found to release mixtures of shorter chain oligosaccharides, including cellotriose, cellobiose and small amounts of glucose (Fig. 2B). Similar results were observed when the substrate was digested with all of the endoglucanases as a mixture, although in this case more glucose was released. Consistently, however, cellobiose was released as the largest proportion of the end products (Fig. 2B).

It was our prediction that the short chain oligosaccharides are the products of the prolonged (16 hours) hydrolytic reactions, i.e., it was possible that the oligosaccharides released from cellulose hydrolysis were initially longer chain oligosaccharides. Further incubation would then allow the longer chains to be hydrolyzed into the shorter chain oligosaccharides and glucose. To test this hypothesis, short-term hydrolytic reactions were conducted using representatives of the proteins with varying modular structures, including Ra0259 (no signal peptide, no accessory domains), Ra0325 (with signal peptide but no accessory domains) and Ra2535 (with signal peptide and accessory domains). All three enzymes (0.5 μM) released longer chain oligosaccharides (degree of polymerization greater than 3), when incubated with PASC (0.5% w/v), as well as shorter chains (DP less than 4) during the initial time points (Supplementary Fig. S2). As the reaction progressed, the proportion of shorter chain oligosaccharides increased with concomitant decreases in the proportion of the longer chain oligosaccharides (Supplementary Fig. S2).

To estimate the kinetic parameters of the *R. albus* 8 endoglucanases, release of cellobiose equivalents were measured following incubation with increasing concentrations of PASC. In general, the endoglucanase activities did not follow Michaelis-Menten kinetics within the substrate range tested. The kinetic parameters could only be estimated for two of the endoglucanases, Ra0903 and Ra2461, which interestingly had the highest and lowest specific activities on PASC, respectively (Fig. 2A). In agreement with the specific activity data, the estimated turnover number (*k*_cat_) for Ra0903 was 1.7 s^−1^ whereas the value was 0.4 s^−1^ for Ra2461. However, the estimated *K*_m_ of Ra0903 for PASC was four times (5.6 mg/ml) that of Ra2461 (1.3 mg/ml), leading to similar estimated catalytic efficiencies (*k*_cat_/*K*_m_) of ~0.3 mg ml^−1^ s^−1^ for the two enzymes (Supplementary Fig. S3).

### Phylogenetic analysis of GH5 modules of *R. albus* 8 endoglucanases

To determine whether *R. albus* 8 has expanded its cellulose degrading capacity by using the same family of GH5 to evolve different endoglucanases, we conducted a phylogenetic analysis of the GH5 catalytic modules characterized in this study. As shown in Supplementary Fig. S4, the *R. albus* 8 GH5 modules fell within three distinct clusters: two in GH5_1, one in GH5_2 and four in GH5_4. These findings support the concept of expansive and increased diversity of GH5 containing enzymes in order to accommodate glycan diversity.

### Functional analysis of carbohydrate-binding modules

**Figure 3:**
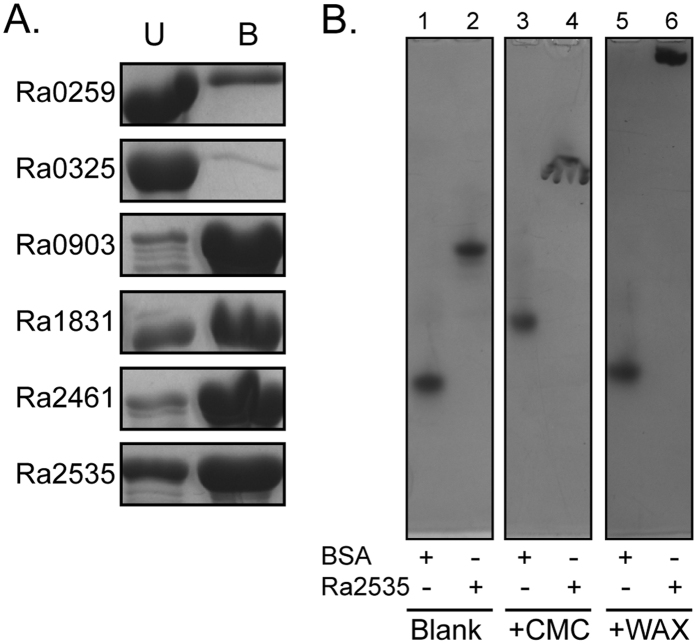
Binding of soluble and insoluble polysaccharides by the *R. albus* 8 endoglucanases. (**A**) Individual endoglucanases (1 mg/ml) were incubated with Avicel (10% w/v) at 4 °C for one hour. The unbound fraction or protein remaining in the supernatant (U) and the bound fraction (**B**) were separated by centrifuging the insoluble cellulose and washing away the proteins that did not adhere to the cellulose pellet. SDS-PAGE analyses and Coomassie staining were used to observe the bound and unbound proteins. Ra0185 was unstable, and therefore it was excluded from the analysis. (**B**) Affinity non-denaturing gel electrophoresis was performed to qualitatively analyze the capacity of Ra2535 to bind to insoluble polysaccharide (Lane 2, 4 and 6). Migration through a non-denaturing gel without polysaccharide (Blank, Lane 1 and 2) was compared to migration through non-denaturing gels containing carboxymethyl-cellulose (CMC, Lane 3 and 4) or wheat arabinoxylan (WAX, Lane 5 and 6). BSA (Lane 1, 3 and 5) was used as a standard for comparison of protein migration.

Putative CBMs are encoded within five of the seven endoglucanases (Fig. 1A). To determine the functions of the CBMs within the polypeptide chain, individual endoglucanases were incubated with Avicel and the bound (B) and unbound (U) protein fractions were determined (Fig. 3A). The endoglucanase Ra0185 was found to be unstable, as several degradation products were always seen during purification. This endoglucanase was, therefore, excluded from the carbohydrate binding analysis. Two of the endoglucanases, Ra0259 and Ra0325, were predominantly found in the unbound fraction, i.e., not associated with cellulose. As these two endoglucanases were not predicted to encode CBMs, it is reasonable that they were unable to bind to the cellulose. The four remaining endoglucanases, Ra0903, Ra1831, Ra2461, and Ra2535 were found predominantly in the bound fraction. Although initially we could not find a CBM of similar sequence with the region now identified as a CBM, subsequent search of the literature indicated that Ra2535 harbors a member of a recently identified CBM family assigned to family 65^11^. So far, this is the only CBM65 homolog identified in the partial genome sequence of *R. albus* 8. The first characterized member of this CBM family was identified in *Eubacterium cellulosolvens*, another cellulose degrading Firmicute found in the rumen^11^. A number of amino acid residues determined to be responsible for binding to substrate in the *E. cellulosolvens* homolog are not present in the CBM65 of Ra2535. Therefore, further binding analysis with other substrates was conducted with Ra2535. Migration of the protein on a non-denaturing polyacrylamide gel and a similar gel infused with carboxymethyl-cellulose, a soluble cellulose derivative, or wheat arabinoxylan, was investigated (Fig. 3B). The CMC infused gel results were inconclusive, as the migration of Ra2535 as well as that of BSA were retarded. However, the migration of Ra2535 was retarded on WAX compared to the non-denaturing gel without substrate, while BSA was not, indicating that the polypeptide binds to the hemicellulose WAX. Interestingly, when Ra2535 was tested for hydrolytic activity against WAX, there was release of reducing ends (data not shown), suggesting that the enzyme also has hydrolytic activity on the xylan portion of the plant cell wall.

### Truncational mutants reveal substrate binding range for the CBM65 of Ra2535 and a novel CBM in *R. albus* 8 Ra1831

**Figure 4:**
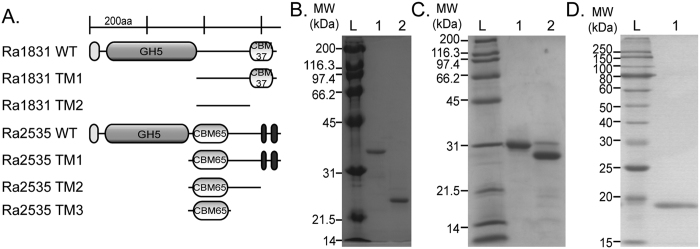
Truncational analyses for identification of CBMs in Ra1831 and Ra2535. (**A**) Domain architecture of the truncational mutants created from Ra1831 and Ra2535. Coloring and domain predictions were performed as in Fig. 1. Abbreviations: CBM, carbohydrate binding module; GH, glycoside hydrolase. (**B**) SDS-PAGE analysis of truncational mutants of Ra1831 purified using cobalt affinity chromatography and gel filtration. Lane L, molecular mass markers; Lane 1, Ra1831 TM1; Lane 2, Ra1831 TM2. (**C**) SDS-PAGE analysis of truncational mutants of Ra2535 purified using cobalt affinity chromatography and gel filtration. Lane L, molecular mass markers; Lane 1, Ra2535 TM1; Lane 2, Ra2535 TM2. (**D**) SDS-PAGE analysis of truncational mutants of Ra2535 CBM65 purified using cobalt affinity chromatography and gel filtration. Lane L, molecular mass markers; Lane 1, Ra2535 TM3.

To further explore the range of substrates bound by the CBM65 of Ra2535, truncational mutants were made from the polypeptide. The endoglucanase Ra1831 also contains a large region without known function, while also possessing a sequence homologous to CBM37. The regions of interest in the two endoglucanases were either cloned alone or with their C-terminal sequences. In Fig. 4A, the modular architectures of the wild-type proteins and the truncational mutants are presented, and the recombinant proteins were purified to near homogeneity (Fig. 4B–D).

Detailed characterization of the Ra2535 truncational mutants were performed, including binding of Ra2535 TM3 to insoluble substrates (Supplementary Fig. S5). As expected, the protein was able to bind tightly to the crystalline cellulose Avicel. Although Ra2535 TM3 shows homology to the CBM65 from *E. cellulosolvens* (EcCBM65), the Ra2535 TM3 protein was also able to bind weakly to mannan and arabinan. These results differed from the results of the binding studies with the *E. cellulosolvens* CBM65. Several key residues identified in the *E. cellulosolvens* CBM65 differ from the CBM found in *R. albus* 8. The residues essential to the binding activity of EcCBM65 include W55, Q106, and Q110. In Ra2535, a serine corresponds to W55 and a lysine corresponds to Q110, while there is a gap in alignment corresponding to Q106 (Supplementary Fig. S6A). Alignment of EcCBM65 and the Ra2535 TM3 with similar sequences, retrieved from the Genbank database, suggests that there are related CBMs yet uncharacterized. Binding to soluble substrates was explored using electrophoresis with non-denaturing affinity gels infused with soluble polysaccharides. As observed for the *E. cellulosolvens* CBM65, the TM3 of *R. albus* 8 was able to bind tightly to xyloglucan and soluble wheat arabinoxylan and lichenin, although we observed that there was also some retardation in migration of the BSA used as the control in this experiment. Weaker binding was identified for konjac glucomannan (KJM), and no binding was seen for galactan and laminarin (Supplementary Fig. S7), while the results for binding to CMC were inconclusive.

Isothermal titration calorimetry was also conducted (Supplementary Table S2) on Ra2535 TM1 and TM2. The affinity constants of Ra2535 TM1 and TM2 for cellopentaose were similar, i.e., 1.68 × 10^5^ M^−1^. The identical affinity constants of Ra2535 TM1 and TM2 reveal the presence of a single region responsible for binding to the oligosaccharide. The number of cellopentaose molecules bound (n) during each binding event was approximately 1 for Ra2535 TM1 and TM2, respectively, suggesting a single binding site within each peptide for cellopentaose. As Ra2535 TM1 and TM2 had the same affinity for cellopentaose, the comparative analysis between cello- and xylopentaose was conducted using Ra2535 TM2 alone. The binding affinity of Ra2535 TM2 for xylopentaose was approximately 15-fold lower than that for cellopentaose. Thus, the ITC results were in agreement with the values obtained for the EcCBM65^11^.

**Figure 5:**
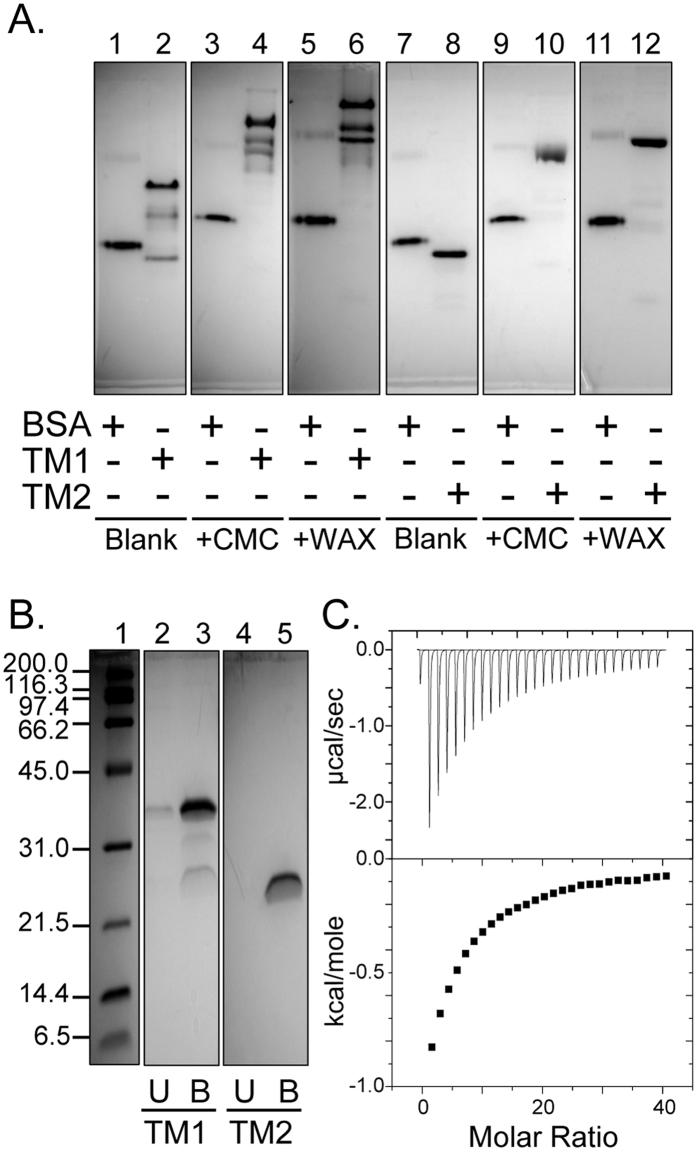
Identification of a novel CBM in Ra1831. (**A**) Affinity non-denaturing PAGE (6%) analysis that compared the migration of Ra1831 TM1 and TM2 in the absence (lanes 2 and 8, respectively) or presence of carboxymethyl cellulose (CMC, lanes 4 and 10, respectively) and wheat arabinoxylan (WAX, lanes 6 and 12, respectively). BSA (lanes 1, 3, 5, 7, 9, and 11) was used as control. (**B**) SDS PAGE (12%) showing the unbound (U) and bound (**B**) fractions of Ra1831 TM1 and TM2 after incubation of 1 mg/ml of protein with 10% w/v of Avicel for one hour at 4 °C with shaking. In Lane 1 are the molecular mass markers. (**C**) Representative binding isotherms of ITC of Ra1831 TM2 (50 μM) with cellopentaose as the substrate at 25 °C.

Ra1831 also contains a large region with no sequence homology to known modules in plant cell wall polysaccharide degrading enzymes. In order to determine whether this region also binds to polysaccharides, a set of truncational mutants was made and tested for their affinity for polysaccharides and cellopentaose. In Fig. 5A, non-denaturing PAGE was used to determine whether the truncational mutants bind to CMC or WAX. Here also BSA was used as a control. When compared to gels without polysaccharides, the CMC infused gel retarded the migrations of Ra1831 TM1 and TM2. The WAX-infused gels displayed similar results. However, these results were not convincing since retardation was also observed for the BSA control in the presence of the soluble substrates. To determine if the mutants are capable of binding to insoluble cellulose, Ra1831 TM1 and TM2 were incubated with crystalline cellulose, and the bound (B) and unbound (U) fractions were compared. In Fig. 5B, the resulting SDS-PAGE gels are shown. Both the Ra1831 TM1 and TM2 bound to the crystalline cellulose (Fig. 5B lanes 3 and 5) revealing that the unknown region within Ra1831 binds to insoluble cellulose. To obtain quantitative information on the new CBM, ITC was performed for both truncational mutants, with cellopentaose as the substrate. Figure 5C shows a representative ITC binding isotherm for Ra1831 TM2. The representative ITC data for Ra1831 TM1 was not very different from that of TM2 (results not shown). In each case, the binding was weak and binding parameters were not deducible from the ITC results. The ITC analysis for Ra1831 TM2 with xylopentaose suggested no binding affinity; however, the binding to cellopentaose, although weak, shows that this region contains a new CBM. The amino acid sequence alignment in Supplementary Fig. S6 shows that this CBM is conserved in endoglucanases found in homologous proteins in *R. albus* strains and also other cellulolytic bacteria.

### Mutational analysis reveal key residues involved in binding of Ra1831 TM2 to substrate

**Figure 6:**
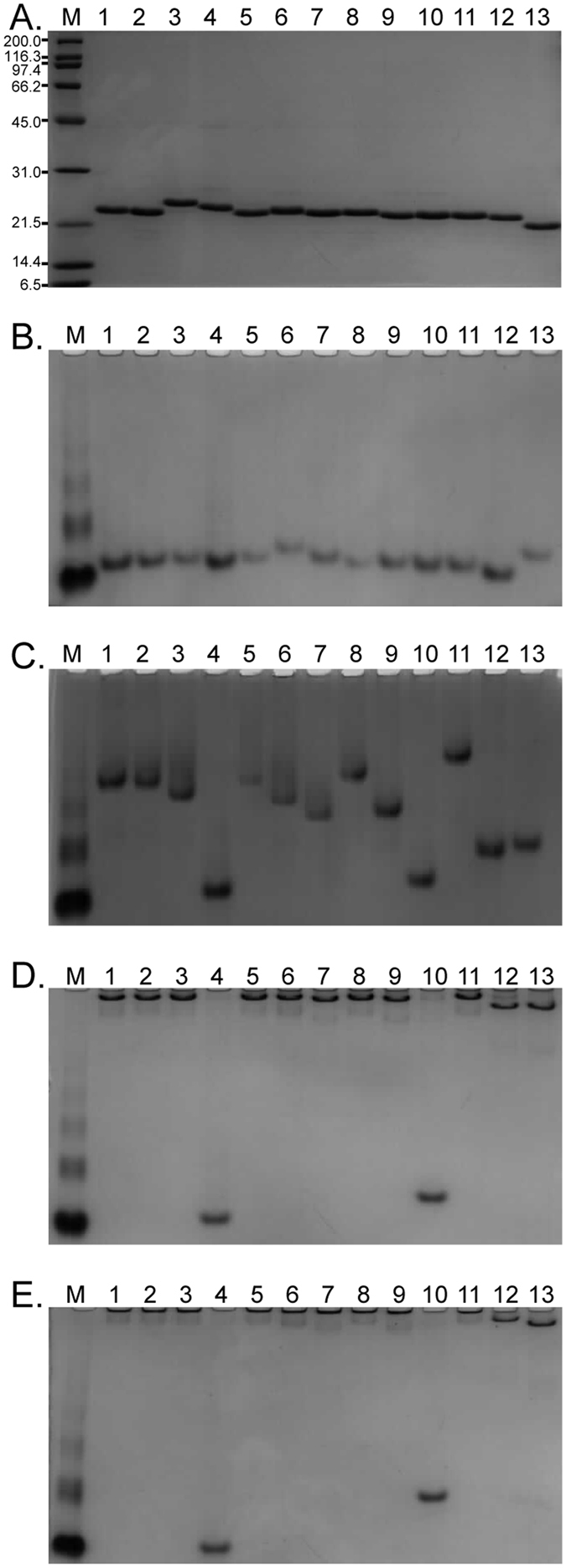
Site directed mutagenesis of Ra1831 TM2 and binding of the mutants to soluble polysaccharides. (**A**) SDS PAGE (12%) of Ra1831 TM2 and its site-directed mutants purified through cobalt affinity chromatography and gel filtration. Abbreviations; Lane M, molecular mass markers. (**B–E**) Affinity non-denaturing PAGE (6%) comparing the migration of Ra1831 TM2 with its single amino acid mutants (2 μg) in the absence (**B**) or presence of soluble polysaccharides (0.2%), including wheat arabinoxylan (**C**), glucomannan (**D**), and xyloglucan (**E**). Abbreviations; Lane M, BSA as migration standard; Lane 1, Ra1831 TM2 WT; Lane 2, Ra1831 TM2 W433A; Lane 3, Ra1831 TM2 Y470A; Lane 4, Ra1831 TM2 W488A; Lane 5, Ra1831 TM2 Y507A; Lane 6, Ra1831 TM2 D509A; Lane 7, Ra1831 TM2 T532A; Lane 8, Ra1831 TM2 Y542A; Lane 9, Ra1831 TM2 Y437A; Lane 10, Ra1831 TM2 W448A; Lane 11, Ra1831 TM2 W456A; Lane 12, Ra1831 TM2 Q481A; Lane 13, Ra1831 TM2 D482A.

Due to the low sequence homology of Ra1831 TM2 to the characterized CBM65, we conducted site-directed mutagenesis to determine whether substrate binding is mediated through the same amino acid residues demonstrated for EcCBM65. The *R. albus* 8 Ra1831 TM2 was aligned with EcCBM65 and the aromatic residues that were conserved in the two proteins were mutated to alanine. A second alignment was constructed between Ra1831 TM2 and Ra2535 CBM65 and amino acids that were conserved were mutated to alanine in Ra1831 TM2. The mutations made included the following: W433A, Y470A, W488A, Y507A, D509A, T532A, Y542A, Y437A, W448A, W456A, Q481A, and D482A (Supplementary Fig. S6). Mutants were expressed and purified to homogeneity in parallel with the non-mutated Ra1831 TM2 (Fig. 6A). To analyze the binding capacity of the mutated derivatives of Ra1831 TM2, protein migration through non-denaturing polyacrylamide gels with and without infusion of substrates, including WAX, glucomannan, and xyloglucan, were determined (Fig. 6B–E). The non-mutated Ra1831 TM2 and all of the mutants migrated to a similar position in the non-denaturing gel without substrate (Fig. 6B). The migrations on non-denaturing gels infused with glucomannan (Fig. 6D) and xyloglucan (Fig. 6E) by Ra1831 TM2 and 10 of the mutants were clearly retarded. However, the mutants W448A and W488A were impaired in their capacity to bind to both substrates and also to wheat arabinoxylan (Fig. 6C). In addition, the mutants Y437A, Y470A, Q481A, D482A, D509A, and T532A also showed some defective binding to WAX (Fig. 6C).

## Discussion

*R. albus* is a major plant cell wall degrading bacterium residing in the rumen. To increase our understanding of the mechanism of cellulose deconstruction by this prominent ruminant microbe, genes predicted to encode endoglucanases were cloned and expressed, and their gene products screened for cellulolytic activity. The polypeptides capable of cellulose hydrolysis were further biochemically characterized.

Typical endoglucanases cleave amorphous regions within cellulose and release long chain cello-oligosaccharides^18^. The endoglucanases in this study were able to release shorter products including glucose, cellobiose and cellotriose from cellulosic substrates. This degradation strategy supports the documented substrate preference for *R. albus* B199, which preferentially ferments cellobiose or cellodextrins over glucose^19^. Cellobiose phosphorylase (EC 2.4.1.20) activity, where cellobiose is cleaved using phosphate instead of water, producing glucose-1-phosphate and glucose or cello-oligosaccharides (DP = n − 1), has been detected in the cytoplasm of *R. albus* B199. The activity of the *R. albus* B199 cellobiose phosphorylase was four fold higher in cells grown on cellodextrins and cellobiose versus glucose^20^. Using the cellobiose phosphorylase eliminates the use of one ATP in glycolysis, by bypassing hexokinase and harnessing phosphoglucomutase to convert glucose-1-phosphate to glucose-6-phosphate.

The putative endoglucanases of *R. albus* 8 exhibited diverse modular architectures. Over half of the endoglucanases contained CBMs that are located C-terminally to the catalytic domain. In contrast, Ra2461 contained a CBM that was N-terminal to the GH catalytic domain. Although the specific activity of this enzyme was the lowest, kinetic parameters showed the estimated catalytic efficiency to be similar to that of Ra0903, the endoglucanase with the highest specific activity. We postulate that the endoglucanases characterized in the present study enhance cellulose deconstruction through their diverse domain architectures.

To investigate the phylogenetic relationship among the GH5 domains within the *R. albus* 8 endoglucanases, a phylogenetic tree was constructed using sequences from the GH5 catalytic domains of the seven enzymes, in addition to representative sequences from all of the GH5 subfamilies used for a similar analysis by Aspeborg *et al.*^21^. The enzymes from this study clustered with three different subfamilies. The GH5_1 subfamily is known to contain primarily endo-β-1,4-glucanases and clustered with the two *R. albus* 8 endoglucanases, Ra0903 and Ra2461. The largest reported subfamily, GH5_2, clustered with only one *R. albus* 8 endoglucanase, Ra0259, which lacks a signal peptide and is not multimodular, as reported for many members of this subfamily^21^. The GH5_4 subfamily is a versatile group of enzymes that includes enzymes that cleave xyloglucan, lichenin and xylan. The *R. albus* 8 enzymes clustering with this family are also promiscuous in their substrate specificities, since Ra0185, Ra0325, Ra1831, and Ra2535 exhibited the ability to hydrolyze diverse glycan substrates including xyloglucan and glucomannan (Table 1).

The endoglucanases of *R. albus* 8 were compared to those of other fibrolytic ruminal bacteria. A prominent cellulolytic bacterium *Fibrobacter succinogenes* S85 harbors many glycoside hydrolases for cellulose deconstruction. Similar to *R. albus* 8, *F. succinogenes* S85 contains enzymes from GH5_4 and GH5_2. There are four members of each of the two subfamilies, including Cel5H within GH5_2. Previous reports describe synergy between FsCel5H and other endoglucanases including Cel9B, Cel8B, and Cel51A^22^. Based on our analysis of the draft genome sequence, *R. albus* 8 also contains homologous genes encoding GH8 and GH51 proteins, suggesting an analogous cellulose degradation strategies may have evolved in the two prominent cellulolytic ruminal bacteria.

Some bacteria within the genus *Prevotella* and *Bacteroides* are well known fibrolytic organisms in gut environments, and they function primarily in hemicellulose degradation. The prominent human gut bacterium, *Bacteroides ovatus* ATCC 8483, contains a single homolog (30% identity) of the gene encoding Ra0325. This gene is located within a polysaccharide utilization locus (PUL) identified as a xyloglucan degradation locus^23^. *Prevotella bryantii* B_1_4, from the rumen, contains two homologs (36 and 34% identity) of Ra0325 and a single GH9 (EFI71705, EFI72295,and EFI73198, respectively). However, these genes are not localized in a defined PUL, unlike in *B. ovatus*. In both bacteria (*B. ovatus* and *P. bryantii*), the homologous GH5 enzymes lack CBMs, which likely minimizes their versatility in plant cell polysaccharide degradation.

A well-known cellulolytic soil bacterium also belonging to the phylum Firmicutes, *Clostridium thermocellum* ATCC 27405, is known for expressing a large multi-protein complex, the cellulosome. The *C. thermocellum* cellulosomes degrade cellulose as well as hemicellulose. The *C. thermocellum* homologs of the *R. albus* 8 GH5_4 proteins include CelE and CelH, and the homologs of the *R. albus* 8 GH5_1 proteins include the *C. thermocellum* CelG, CelB, CelO, and a predicted endoglucanase (accession number CAK18680). The homologous enzymes of *C. thermocellum* lack CBMs, but all contain dockerin domains for incorporation into the cellulosome. The *C. thermocellum* CelO encodes a cellobiohydrolase and contains a CBM3 at the N-terminus, a modular organization similar to Ra2641, which has a CBM2 at the N-terminus^24^. The GH5_1 domains of Ra2461 and CtCelO share 49% amino acid sequence identity.

Carbohydrate-binding modules are commonly found within endoglucanases, as they improve affinity of an enzyme for insoluble substrates, essentially increasing enzyme concentration on the surface of the cellulose microfibril^8^,^10^. The lifestyle of *R. albus* 8 requires this bacterium to adhere to insoluble plant material in order to hydrolyze and ferment the cellulose and hemicellulose. The extracellular enzymes used by *R. albus* 8 are thus expected to possess CBMs to ensure fitness in the rumen. There are a total of nine CBMs encoded within the *R. albus* 8 endoglucanases biochemically characterized in this study. These cellulose-binding CBMs fall within four different families (2, 4, 37, and 65). In addition, three of these families (4, 37, and 65) of CBMs are known to bind to xylan. Thus, these diverse CBMs likely ensure binding of the endoglucanases to the major polysaccharides present in plant cell walls, the main energy source for ruminants.

Due to the variety and complex arrangements of CBMs, the unidentified region within the endoglucanase Ra1831 was hypothesized to contain a new CBM. The truncational analysis that delineated the unknown regions in the polypeptides identified these modules as encoding CBMs capable of binding to cellulosic oligosaccharides. The CBM from Ra2535 has 24% amino acid sequence identity to the recently identified member of CBM family 65. The CBM65 from *E. cellulosolvens* has been characterized and key amino acid residues have been identified^11^. Through a comprehensive screening of polysaccharide substrates, the Ra2535 CBM65 was also shown to bind to a wide range of polysaccharides including mannan and arabinan (Supplementary Fig. S5). The differences in some of the key residues may account for the differences in substrate recognition between the *R. albus* 8 CBM65 and its homolog in *E. cellulosolvens*^11^. Mutational analysis revealed two residues (W448 and W488) already shown to be essential in EcCBM65 as also critical in Ra1831 TM2, hence providing evidence for a similar binding mechanism in the *R. albus* CBM65. In addition, we uncovered a novel CBM (Ra1831 TM2) in this study. The new CBM does not show significant sequence homology to other characterized proteins in the NCBI database. We anticipate that as more genome sequences of carbohydrate/polysaccharide degrading organisms become available in the future, the sequence of the new CBM may be used to identify novel carbohydrate active enzymes to which it is anchored.

In a previous report, we identified and presented the contribution of several enzymes that enable *R. albus* 8 to completely deconstruct complex hemicellulose^25^. In the present work, we present biochemical evidence for the mechanism that is likely used by *R. albus* 8 to initiate depolymerization of cellulose. We look forward to characterizing the necessary enzymes that lead to further degradation of the oligosaccharides released by the *R. albus* 8 endoglucanases. In addition to enhancing our understanding of plant cell wall degradation by a prominent ruminal bacterium, the results from this work are also important to the prospecting of enzymes of biomass degradation for biofuel production.

## Methods

### Materials

Genomic DNA was extracted from a culture of *R. albus* 8 stored in a culture collection at the Department of Animal Science at the University of Illinois at Urbana-Champaign. *Escherichia coli* strains, JM109 and BL-21-CodonPlus (DE3) RIPL, were purchased from Stratagene (La Jolla, CA). The pET-46 Ek/LIC vector kit was from Novagen (San Diego, CA). The pGEM-T Easy Vector system was purchased from Promega (Madison, WI). QIAprep Spin Miniprep kit was from Qiagen (Valencia, CA). Cello-oligosaccharides and medium viscosity wheat arabinoxylan (WAX) were purchased from Megazyme (Bray, Ireland). All other reagents were of the highest possible purity and were purchased from Sigma-Aldrich (St. Louis, MO).

### Gene cloning

A bioinformatic search of the *R. albus* 8 draft genome sequence (accession no. NZ_ADKM00000000) revealed 17 genes as putatively involved in cellulose degradation. These genes include two open reading frames (ORFs) for putative GH3 β-glucosidases (*ra2003* and *ra3307*) and three ORFs coding for putative GH9 exoglucanases (*ra2259, ra3055, ra3241*). There were 12 genes that were predicted to encode endoglucanases; ten GH5 (*ra0185*, *ra0259*, *ra0325*, *ra0711*, *ra0903*, *ra1553*, *ra1831*, *ra2461*, *ra2535*, *ra2979*), one GH9 (*ra2876*), and one GH48 (*ra2561*) polypeptides.

The genes for the putative endoglucanases were cloned using primers designed to delete the N-terminal region coding for signal peptides to prevent secretion and rather enhance accumulation of the recombinant protein in the cell (Supplementary Table S3). Signal peptides were predicted using the SignalP v3.0 online server (www.cbs.dtu.dk/services/SignalP/) (Supplementary Table S3).

Each gene analyzed in this study was amplified using the PicoMaxx high fidelity PCR kit (Stratagene) and cloned into the pET-46b vector using the ligation independent cloning technique following the protocols of the manufacturer. Recombinant plasmids were transformed into *E. coli* JM109 cells via electroporation, plated, and grown for 16 hours at 37 °C on lysogeny broth (LB) agar plates supplemented with ampicillin (100 μg/mL). A single colony from each transformation was inoculated into LB medium (10 mL) containing ampicillin (100 μg/mL) and grown to saturation. The cells were subsequently centrifuged (3220 × g, 15 min, 4 °C) and plasmids were extracted from the resulting cell pellet using the QIAprep Spin Miniprep kit (Qiagen, Valencia, CA). The foreign DNA inserts in the recombinant plasmids were sequenced to confirm the correctness of the gene sequence (W. M. Keck Center for Comparative and Functional Genomics at the University of Illinois at Urbana-Champaign). Ra2876 was unable to be cloned and excluded from further analysis.

### Gene expression and protein purification

For gene expression, the pET46b vector with the correct DNA insert was transformed into *E. coli* BL-21 CodonPlus (DE3) RIPL cells by the heat shock method and grown overnight at 37 °C on LB agar plates supplemented with ampicillin (100 μg/mL) and chloramphenicol (50 μg/mL). After 16 hours, five colonies were used to inoculate 10 mL of fresh LB medium supplemented with 100 μg/mL of ampicillin and 50 μg/mL of chloramphenicol and grown at 37 °C for 6–8 hours with vigorous aeration. The pre-cultures were then added to fresh LB medium (1 L), supplemented with the two antibiotics at the same concentrations as described above, and grown with vigorous aeration at 37 °C. At OD_600_ of 0.3, isopropyl β-D-thio-galactopyranoside (IPTG) was added to each culture at a final concentration of 0.1 mM, and the temperature was decreased to 16 °C. Following 16 hours of culturing, cells were pelleted by centrifugation (4,000 × g, 15 min, 4 °C) and re-suspended in 30 mL of binding buffer (50 mM Tris-HCl, 300 mM NaCl, pH 7.5). The cell suspensions were subjected to two passages through an EmulsiFlex C-3 cell homogenizer (Avestin, Ottawa, Canada), and the cell debris in the cell lysates were removed by centrifugation (20,000 × g, 30 min, 4 °C).

To purify the recombinant proteins, Talon Metal Affinity Resin (Clontech) was used according to the protocol of the manufacturer with a modified binding/wash buffer (50 mM Tris-HCl, 300 mM NaCl, pH 7.5) and elution buffer (50 mM Tris-HCl, 300 mM NaCl, 150 mM imidazole, pH 7.5). Ra2561 was found to be insoluble and excluded from further analysis. Partially purified proteins were screened for catalytic activity by spotting 5 μL of protein to a substrate infused (0.25%) agarose petri dish containing either CMC, glucomannan, or xyloglucan. The plate was incubated at 37 °C for 16 hours and subsequently stained with Congo Red and destained with 1 M NaCl. Thin layer chromatography was also employed to screen for activity as described below (Supplementary Fig. S1).

To further purify the seven proteins capable of degrading cellulose, elution fractions from the Talon resin containing Ra0185, Ra0259, Ra0325, Ra0911, Ra1831, Ra2461 or Ra2535 were subjected to size-exclusion chromatography (HiLoad 16/60 Superdex 200 prep grade column, GE Healthcare) on an AKTAxpress fast protein liquid chromatograph equipment (FPLC, GE Healthcare). The chromatography was developed with a Tris buffer (50 mM Tris-HCl, pH 7.0). Elution fractions of Ra0259 and Ra0325 were dialyzed into protein storage buffer (50 mM Tris, 150 mM NaCl, pH 7.5) and stored at 4 °C until used for enzymatic reactions. For Ra0185, Ra0911, Ra1831, Ra2461, and Ra2535, anion exchange chromatography (5 mL HiTrap Q HP column, GE Healthcare) was performed after gel filtration to achieve purification. Proteins were bound to the column in the same buffer used for gel filtration, and eluted with the binding buffer supplemented with 1 M NaCl (50 mM Tris-HCl, 1 M NaCl, pH 7.0). Elution fractions were pooled and dialyzed into protein storage buffer and stored at 4 °C until used for enzymatic reactions. Sodium dodecyl sulfate-polyacrylamide gel electrophoresis (SDS-PAGE) was used to assess protein purity. The protein bands were visualized by staining with Coomassie brilliant blue G-250, as described previously^26^. Purified proteins were quantified by measuring absorbance at 280 nm (A_280nm_) using a NanoDrop 1000 (Thermo Scientific, Waltham, MA) and the molecular mass and the theoretical extinction coefficient of each protein (listed in Supplementary Table S3).

### Analysis to assign function to regions of unknown function in the *R. albus* 8 endoglucanases

To identify the function of unknown regions of Ra1831 and the CBM65 from Ra2535, truncational mutants were produced and tested for carbohydrate binding activity. The *R. albus* 8 Ra1831 TM1 contained approximately the C-terminal half of the entire polypeptide, including the region of unknown function as well as the CBM37. The TM2 truncational mutant contained only the region of unknown function. The Ra2535 TM1 truncational mutant contained the C-terminal half of the polypeptide, including the putative CBM65 and dockerin domains. Compared to the TM1 mutant, the Ra2535 TM2 mutant lacked the dockerin-like sequences, and the TM3 contained only the putative CBM65. The primers used to create the truncated derivatives are shown in Supplementary Table S3. The DNA sequences encoding the regions of unassigned function in Ra1831 (or TM1 and TM2) were cloned into a modified pET-28a vector as described previously^27^. Briefly, primers were designed to include a 5′-NdeI and 3′-XhoI restriction sites to facilitate cloning (Supplementary Table S3). After amplification using PicoMaxx high fidelity PCR kit, the PCR products were ligated into the pGEM-T vector following the protocol of the manufacturer. The inserts were excised from the plasmids by digesting with NdeI and XhoI and then ligated into a modified pET-28a vector already digested with NdeI and XhoI. The inserts in the recombinant plasmids were sequenced to confirm the correctness of each DNA insert (W. M. Keck Center for Comparative and Functional Genomics at the University of Illinois). The expression and purification of Ra1831 TM1 and TM2 were carried out as described above for the full length protein. The regions of Ra2535 designated TM1, TM2, and TM3 were cloned, expressed, and the polypeptides purified as described above for the full length protein. Final protein concentrations were calculated using the molecular mass and theoretical extinction coefficients (listed in Supplementary Table S4).

### Mutational analysis of Ra1831 TM2 to identify key residues for binding to substrate

In order to identify amino acid residues that may be involved in binding to substrates, an amino acid sequence alignment was carried out with Ra1831 TM1 and its related sequences in the NCBI. Mutations were made in the pET-46b containing the non-mutated Ra1831 TM2 using QuikChange Lightning Site-Directed Mutagenesis Kit (Agilent, Santa Clara) with the primers listed in Supplementary Table S5. Mutated plasmids were transformed into *E. coli* JM109 by electroporation and grown overnight at 37 °C on LB agar plates supplemented with ampicillin (100 μg/mL). Individual colonies from each mutagenic PCR were inoculated into LB, with ampicillin, and plasmids were extracted using the QIAprep Spin Miniprep kit (Qiagen, Valencia, CA). Plasmids containing the expected mutations were identified by DNA sequencing, and the proteins were expressed and purified using the protocol described above for Ra1831 TM2. Each mutated derivative of Ra1831 TM2 was purified by immobilized metal affinity chromatography (Talon resin) and gel filtration with the buffers described above for the two methods. The proteins were quantified based on their molecular mass and theoretical extinction coefficient as listed in Supplementary Table S5.

### Determination of specific activities of *R. albus* 8 endoglucanases on phosphoric acid swollen cellulose (PASC)

The specific activities of Ra0185, Ra0259, Ra0325, Ra0903, Ra1831, Ra2461, and Ra2535 were determined at 37 °C in a buffer composed of 50 mM sodium phosphate (pH 6.5) and 150 mM NaCl. Phosphoric acid swollen cellulose (PASC) was prepared following the method of Wood^28^. Each protein (0.5 μM of Ra0185, Ra0325, Ra0903, Ra1831, Ra2461, and Ra2535 and 1.0 μM of Ra0259) was incubated with PASC (0.5% w/v, final concentration) for 0, 3, 6, and 9 min, and the samples were boiled to stop the reaction. The concentration of soluble reducing ends released was quantified with the *para*-hydroxybenzoic acid hydrazide (PAHBAH) method as described previously^29^. Cellobiose, representing the repeating unit of cellulose, was used as the standard in generating a prediction equation to estimate the reducing ends released by each enzyme on the PASC. Specific activity was calculated as the amount of cellobiose equivalent released per time per enzyme concentration.

Kinetic parameters were estimated using a range of PASC concentrations from 0.08% w/v to 1.2% w/v. This range of substrate concentration was used due to a lack of sensitivity below 0.08% w/v and concentrations above 1.2% w/v were too viscous. The methods were described in our previous reports^30^,^31^.

### End products from hydrolysis of phosphoric acid swollen cellulose (PASC)

Hydrolysis of PASC by Ra0185, Ra0259, Ra0325, Ra0903, Ra1831, Ra2461, and Ra2535 were determined by incubating each enzyme (0.5 μM) with PASC (0.5% w/v, final concentration) in the reaction buffer (50 mM sodium phosphate, 150 mM NaCl, pH 6.5) for 16 hours at 37 °C. The resulting concentration of reducing ends was quantified using the PAHBAH method with the standard curve generated as described above. Thin layer chromatography (TLC) was employed to identify the products of hydrolysis from PASC. The TLC (plate dimensions 10 cm × 20 cm) method followed the procedure described in our earlier report^25^.

### Quantitative analysis of end products of hydrolysis

To quantitatively assess products released from the enzymatic hydrolysis, high performance anion exchange chromatography (HPAEC) was used to separate the mono- and oligo-saccharides produced from substrate hydrolysis. The methods were as described in our previous reports^30^,^31^. The identity and concentration of mono- and oligo-saccharides produced from enzymatic hydrolysis of polysaccharides were determined by comparison of peak retention times and peak areas to those of known oligosaccharides with known concentrations.

### Binding assays

To determine the functions of the putative carbohydrate-binding modules within Ra0185, Ra0903, Ra1831, and Ra2461, each enzyme (1 mg/ml) was incubated with Avicel (10% w/v) for 1 hour at 4 °C. After the incubation, each reaction mixture was centrifuged (25,000 × g, 10 minutes, 4 °C) to pellet the Avicel and any enzyme or protein bound to the model crystalline cellulose. The residual proteins (present in the supernatant) were analyzed as the unbound fraction for comparison. After three washes in the reaction buffer (50 mM sodium phosphate, 150 mM NaCl, pH 6.5), the protein associated with the pelleted Avicel was eluted by boiling with SDS-buffer and designated the bound fraction. The bound and unbound fractions (protein remaining in solution) were visualized via SDS-PAGE. During analysis of Ra0185, the SDS-PAGE analysis found only a small portion of Ra0185 with the correct size, but several bands corresponding to cleaved or degraded products (data not shown), potentially due to the prolonged incubation with Avicel. Therefore, this protein was omitted from this analysis.

To determine the binding of recombinant proteins to soluble polysaccharides, non-denaturing gel electrophoresis was used. Polyacrylamide gels (6%) were infused with carboxymethyl cellulose (CMC) or wheat arabinoxylan (WAX) at a concentration of 0.25% w/v. Each recombinant protein or bovine serum albumin (BSA as control) (2 μg) was loaded into wells and electrophoresed for 2 hours and 15 minutes at 80 V. To visualize the protein bands, the gels were stained with Coomassie brilliant blue G-250. The migration and banding patterns were compared between carbohydrate-infused gels and gels without substrate electrophoresed in juxtaposition.

### Analysis of substrate binding of mutant CBMs by Isothermal titration calorimetry (ITC)

Isothermal titration calorimetry was performed at 25 °C using a VP-ITC micro-calorimeter (MicroCal Inc., Northampton, UK). Each of the Ra2535 and Ra1831 truncational mutants was diluted to 50 μM in reaction buffer (50 mM sodium phosphate, 150 mM NaCl, pH 6.5) and 28 successive injections of 10 μl of oligosaccharide (10 mM cellopentaose or xylopentaose in reaction buffer) were administered at 300 second intervals. A non-linear regression, using a single site binding model (MicroCal Origin software), was used to analyze the data collected, and the thermodynamic parameters were calculated with both the Gibbs free energy equation (Δ*G* = Δ*H* *−* *T*Δ*S*) and the relationship Δ*G* = −*RT* ln*K*_*a*_.

### Phylogenetic analysis

Representative GH5 from each subfamiliy, excluding GH5_29, GH5_35, and GH5_50 due to low numbers of representatives, were retrieved and trimmed to contain only the catalytic domain, using dbCAN (csbl.bmb.uga.edu/dbCAN/annotate.php) and Pfam (pfam.sanger.ac.uk/). ClustalW was used to obtain the phylogenetic tree and the circular cladogram was drawn using MEGA6 (www.megasoftware.net/).

## Additional Information

**How to cite this article**: Iakiviak, M. *et al*. Functional and modular analyses of diverse endoglucanases from *Ruminococcus albus* 8, a specialist plant cell wall degrading bacterium. *Sci. Rep.* **6**, 29979; doi: 10.1038/srep29979 (2016).

## Supplementary Material

Supplementary Information

## Figures and Tables

**Table 1:** Screening of *R. albus* 8 putative family 5 endoglucanases for enzymatic activity on different glucans.

	Ra0185	Ra0259	Ra0325	Ra0903	Ra1831	Ra2461	Ra2535
CMC	++^a^	±	++	+	+	+	+
Glucomannan	++	−	++	±	++	+	++
Xyloglucan	++	−	++	±	+	±	++

Enzymes were spotted on agar plate infused with polysaccharide and incubated overnight at 37 °C. Activity was detected by the Congo red assay as described in the manuscript.

^a^(−, No halo; ±, small unclear halo; +, clear halo; ++, large halo).
