# Supplementary material for: Functional and modular analyses of diverse endoglucanases from Ruminococcus albus 8, a specialist plant cell wall degrading bacterium
Source: Sci Rep. 2016 Jul 21;6:29979. doi: 10.1038/srep29979 (PMC4954948; doi:10.1038/srep29979)
Supplement: Supplementary Information [file srep29979-s1.pdf]

Supplementary Information

Functional and modular analyses of diverse endoglucanases from *Ruminococcus albus* 8, a specialist plant cell wall degrading bacterium

Michael Iakiviak[1,2,3], Saravanan Devendran[1,3], Ania Skorupski[1,4], Young Hwan Moon[1,3], Roderick I. Mackie[1,2,3], and Isaac Cann[1,2,3,4,5*]

Energy Biosciences Institute,[1] Department of Animal Sciences,[2] Carl R. Woese Institute for Genomic Biology,[3] School of Molecular and Cellular Biology[4], Department of Microbiology,[5] and University of Illinois at Urbana-Champaign, Urbana, Illinois 61801, USA

Supplemental Tables

SI Table 1. ***R. albus* genes previously cloned, expressed, and demonstrated to exhibit cellulose degrading activity**

Cellulases	Accession #	*R. albus* strain	Secreted (predicted)[a]	GH family[b]	CBM Family[b]
EGI (CelA)	AAA26469	F-40	Yes	GH 5	
EgIV (CelB)	BAA32286	F-40	No	GH 5	
EgV (CelC)	BAA92146	F-40	Yes	GH 5	
Cel5D	BAA92430	F-40	Yes	GH 5	2xCBM4_9
Cel9A[c]	BAB64431	F-40	Yes	GH 9	CBM3
EgA	AAA26467	SY3	No	GH 5	
EgB	CAA38693	SY3	Yes	GH 5	

[a] Signal peptides were predicted using SignalP (http://www.cbs.dtu.dk/services/SignalP/)
[b] Domain identity was predicted using Pfam (http://pfam.sanger.ac.uk/)
[c] Proteins not biochemically characterized

SI Table 2. **Binding parameters of Ra2535 truncational mutants for cello- and xylo-oligosaccharides determined by ITC**

Mutant	Substrate	*N*	K_a x10^3 (M^{-1})	Δ*G* (*kJ*)	Δ*H* (*kJ*)	*T*Δ*S* (*kJ*)	Relative affinity (%)
TM1[a]	Cellopentaose	1.28 ± 0.01	168 ± 6.00	-7.13 ± 0.23	-14.9 ± 0.23	-7.78	100
TM2[b]	Cellopentaose	1.37 ± 0.01	168 ± 6.56	-7.12 ± 0.18	-15.3 ± 0.18	-8.24	100
	Xylopentaose	1.12 ± 0.28	10.1 ± 1.35	-5.46 ± 1.35	-18.3 ± 1.35	-12.39	6

[a] Data from 1 replicate

[b] Data from 3 replicates

SI Table 3. **Primers used in cloning *R. albus* 8 functional endoglucanases, their predicted signal peptides, and characteristic of recombinant proteins.**

Gene	Accession number	Forward primer	Reverse primer	Signal peptide (N → C terminal)	MW (kDa) [a]	Extinction coefficient (M^{-1} cm^{-1}) [a]
ra0185	ZP_08160335.1	5'- GACGACGACAAGATGGCTTT CGAGAAAGATGCCAAACAGACC -3'	5'- GAGGAGAAGCCCGGTTCAGT TGCTCTTTGTGATGACCAAGTCTG -3'	MKTKSRRALTLSTVCAMALSCLAFAEPTRAEA	82.2	148,795
ra0259	ZP_08160389.1	5'- GACGACGACAAGATGCTGAAAA AACTAAAGGTTATCGGAGGAAGG -3'	5'- GAGGAGAAGCCCGGTTCATTTG TTTTTCTCCTCTTCTGCAAAGTCC -3'	No signal peptide	36.7	62,630
ra0325	ZP_08158005.1	5'- GACGACGACAAGATGGATAA GGACAGCAAGACAGAAAGCAGG -3'	5'- GAGGAGAAGCCCGGTTC ATGAAGCGGAAGCAGGGTC -3'	MKLKKFAALFIAAAMSVGTLSACG	44.9	78,965
ra0903	ZP_08159293.1	5'- GACGACGACAAGATGGCAACA TCAGCAGTGAATGACACCAATG -3'	5'- GAGGAGAAGCCCGGTTTACTTTACA GTGATAGTCACAGCGTTCTTGATAGC-3'	MNKVFKRMTAAASVAALTLSFAAASMPAVVTASA	73.5	202,515
ra1831	ZP_08160422.1	5'- GACGACGACAAGATGCTGC CTTCAGCAGTCATCGAAGC -3'	5'- GAGGAGAAGCCCGGTTTACGGAAC TGTAACTATTACAGCGTTCTTGATCG -3'	MKKTIQGRVVSALSAAAIALTGMSSLPSAVIEA	72.0	171,340
ra2461	ZP_08158504.1	5'- GACGACGACAAGATGGAAAAATCTG ACAAAGACGCAGTTTCAAAGAAGTC-3'	5'- GAGGAGAAGCCCGGTTCAAA GCCCCTTAGCTTTTGAAAGCG -3'	MNGGISKKIIAAFVALAAVIIALVVLLVVTNFSGG	70.5	161,145
ra2535	ZP_08158565.1	5'- GACGACGACAAGATGAAAGAC GTGTCAAAAATGACACCCTTCG -3'	5'- GAGGAGAAGCCCGGTTTATCC TGCAATAGTTTTGTCGGGGAGC -3'	MKVTFKRMLSLAAAGAMTLSAMPVLTASA	71.5	120,460

[a] ProtParam (http://web.expasy.org/protparam/) was used to predict molecular weights and extinction coefficients

SI Table 4. **Primers used in cloning truncational mutants of Ra1831 and Ra2535 and characteristic of recombinant proteins.**

Gene	Forward Primer / Reverse Primer	MW (kDa) [a]	Extinction coefficient (M^{-1} cm^{-1}) [a]
ra1831 TM1	5’- **CATATG**GGCTGCACCTGGTATCCTAACTCTG -3’	32.2	80,790
	5’- **CTCGAG**TTACGGAACTGTAACTATTACAGCGTTCTTG -3’		
ra1831 TM2	5’- **CATATG**GGCTGCACCTGGTATCCTAACTCTG -3’	20.6	53,860
	5’- **CTCGAG**TTAGCCTGAAACAGGTACCGCATAC -3’		
ra2535 TM1	5’-GACGACGACAAGATGTCTCTGCCGATGGTAAACAAGCTGATC -3’	34.7	46,300
	5’- GAGGAGAAGCCCGGTTTATCCTGCAATAGTTTTGTCGGGGAGC -3’		
ra2535 TM2	5’- GACGACGACAAGATGTCTCTGCCGATGGTAAACAAGCTGATC -3’	27.2	44,810
	5’- GAGGAGAAGCCCGGTTAACCGCTGATGTCGGCGG -3’		
ra2535 TM3	5’- GACGACGACAAGATGTCTCTGCCGATGGTAAACAAGCTGATC -3’	12.0	10,430
	5’- GAGGAGAAGCCCGGTTAGCCGTAGGCAGACCTGTATG -3’		

Bolded and underlined sequence indicate the NdeI site

Bolded sequence indicate the XhoI site

[a] ProtParam (http://web.expasy.org/protparam/) was used to predict molecular weights and extinction coefficients

SI Table 5. **Primers used in site directed mutagenesis of r*a1831 TM2* and characteristic of recombinant proteins.**

Gene	Forward primer	Reverse primer	MW (kDa)[a]	Extinction coefficient (M^{-1} cm^{-1})[a]
ra1831 TM2 W433A	5’- CGTAAATATAGGTTCTGACGCGGTAGAGCTTTACAAGAATC -3’	5’- GATTCTTGTAAAGCTCTACCGCGTCAGAACCTATATTTACG -3’	20.435	48,360
ra1831 TM2 Y470A	5’- CAAGTTCGTACTCGTTGCCGATTCCGATCAGGCAC -3’	5’- GTGCCTGATCGGAATCGGCAACGAGTACGAACTTG -3’	20.458	52,370
ra1831 TM2 W488A	5’- GCAGCTACGACACAGCAAACCGCGTAGCTTCC -3’	5’- GGAAGCTACGCGGTTTGCTGTGTCGTAGCTGC -3’	20.435	48,360
ra1831 TM2 Y507A	5’- CTTACATAAAGGAATTCTATGCAGATGACCTGACAAGCGCTATC -3’	5’- GATAGCGCTTGTCAGGTCATCTGCATAGAATTCCTTTATGTAAG -3’	20.458	52,370
ra1831 TM2 D509A	5’- GAATTCTATTATGATGCCCTGACAAGCGCTATC -3’	5’- GATAGCGCTTGTCAGGGCATCATAATAGAATTC -3’	20.506	53,860
ra1831 TM2 T532A	5’- CTCTACATCAGCGCTGCCACGAGTTCGCTG -3’	5’- CAGCGAACTCGTGGCAGCGCTGATGTAGAG -3’	20.520	53,860
ra1831 TM2 Y542A	5’- GACTGCTTACGGTCTGGCTGCGGTACCTGTTTC -3’	5’- GAAACAGGTACCGCAGCCAGACCGTAAGCAGTC -3’	20.458	52,370
ra1831 TM2 Y437A	5’- GACTGGGTAGAGCTTGCCAAGAATCCTAACG -3’	5’- CGTTAGGATTCTTGGCAAGCTCTACCCAGTC -3’	20.458	52,370
ra1831 TM2 W448A	5’- GCTCCACCATTGAAGCAGCGAAGAACTTCACAGTATC -3’	5’- GATACTGTGAAGTTCTTCGCTGCTTCAATGGTGGAGC -3’	20.435	48,360
ra1831 TM2 W456A	5’- GAACTTCACAGTATCAAACGCGAAGAACTATGTAAACAAG -3’	5’- CTTGTTTACATAGTTCTTCGCGTTTGATACTGTGAAGTTC -3’	20.435	48,360
ra1831 TM2 Q481A	5’- CACCCGAGCTGGTTCTCGCAGACGGCAGCTACGACAC -3’	5’- GTGTCGTAGCTGCCGTCTGCGAGAACCAGCTCGGGTG -3’	20.493	53,860
ra1831 TM2 D482A	5’- GAGCTGGTTCTCCAGGCAGGCAGCTACGACACATG -3’	5’- CATGTGTCGTAGCTGCCTGCCTGGAGAACCAGCTC -3’	20.506	53,860

[a] ProtParam (http://web.expasy.org/protparam/) was used to predict molecular weights and extinction coefficients

Supplemental Figures

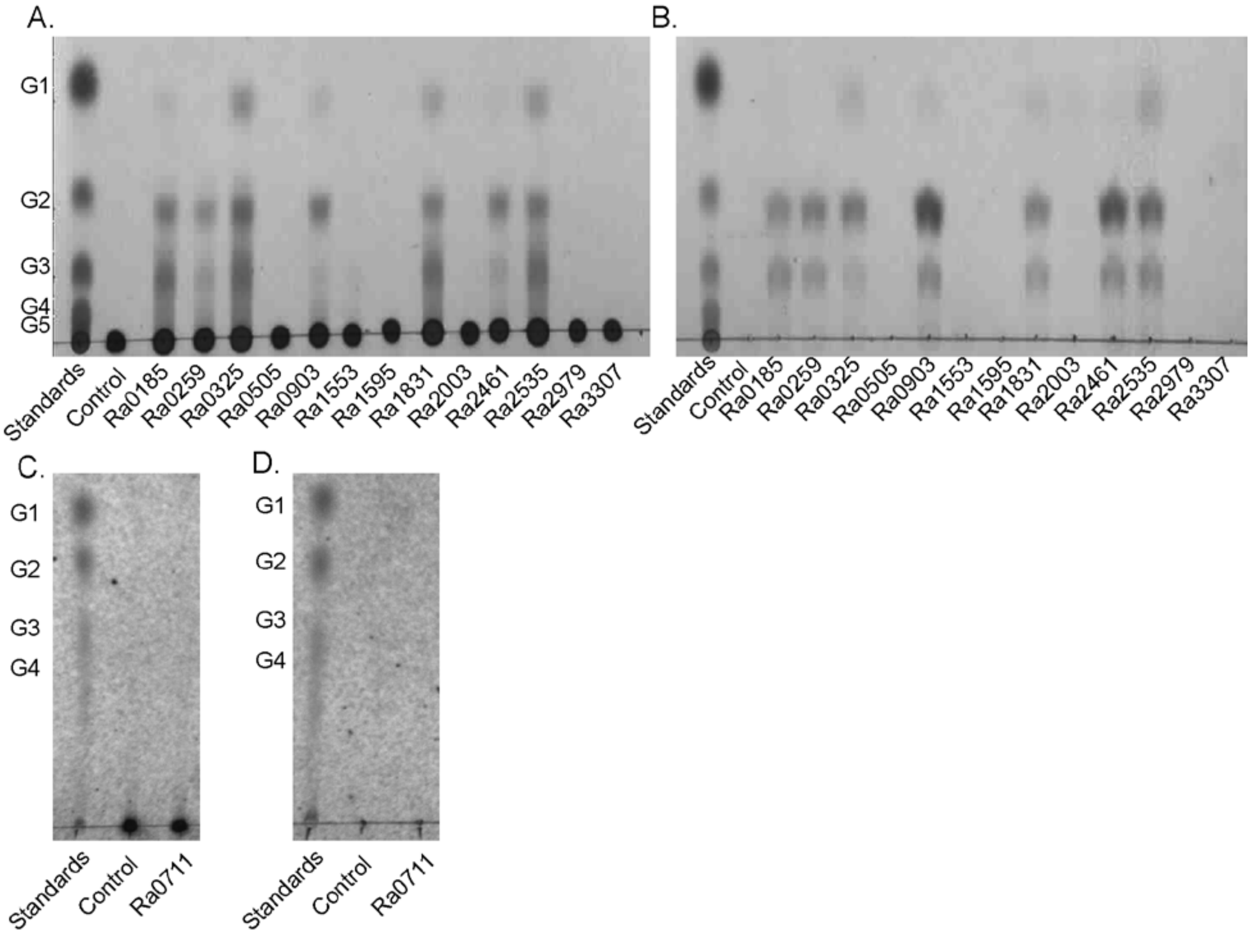

Figure S1. **Thin-layer chromatography screening of *R. albus* 8 endoglucanases**. Cellulosic substrates, CMC (0.5% w/v) (A,C) and PASC (0.5% w/v) (B,D), were incubated with partially purified putative endoglucanases (5% v/v) for 16 hours. The soluble products were separated by thin layer chromatography and visualized by spraying with methanolic orcinol.

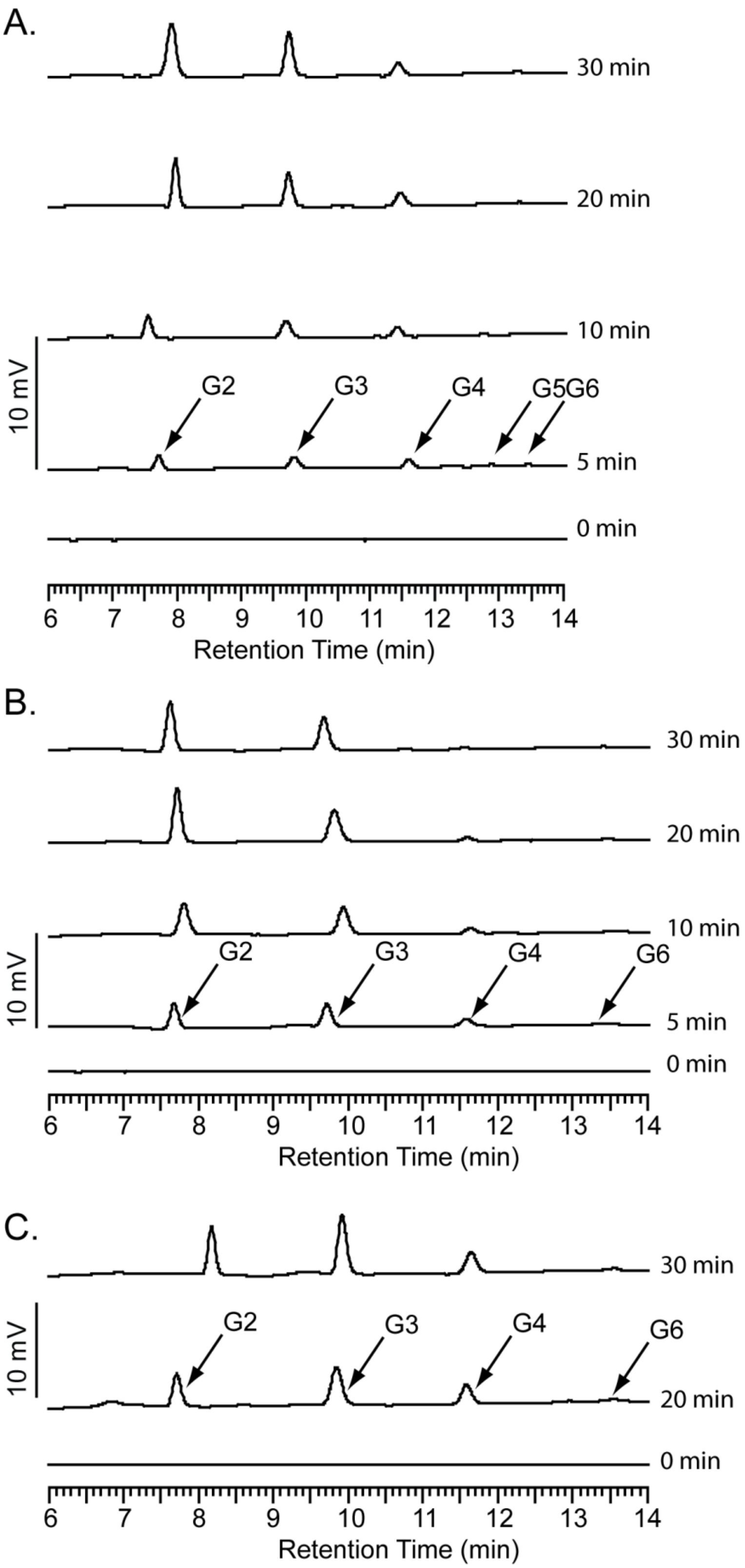
A.
30 min
20 min
10 min
10 mV
G2
G3
G4
G5G6
5 min
0 min
6 7 8 9 10 11 12 13 14
Retention Time (min)
B.
30 min
20 min
10 min
10 mV
G2
G3
G4
G6
5 min
0 min
6 7 8 9 10 11 12 13 14
Retention Time (min)
C.
30 min
10 mV
G2
G3
G4
G6
20 min
0 min
6 7 8 9 10 11 12 13 14
Retention Time (min)

Figure S2. **Time course analysis of hydrolysis of phosphoric acid swollen cellulose by Ra0259, Ra0325 and Ra2535**. PASC was incubated with endoglucanase (0.5 μM) for 0, 5, 10, 20, and 30 minutes. The soluble products were separated and detected using HPAEC-PAD. Representative HPAEC-PAD chromatographs are shown for Ra0259 (A), Ra0325 (B), and Ra2535 (C). Products were identified by comparison of retentions times to those of commercially available cello-oligosaccharides.

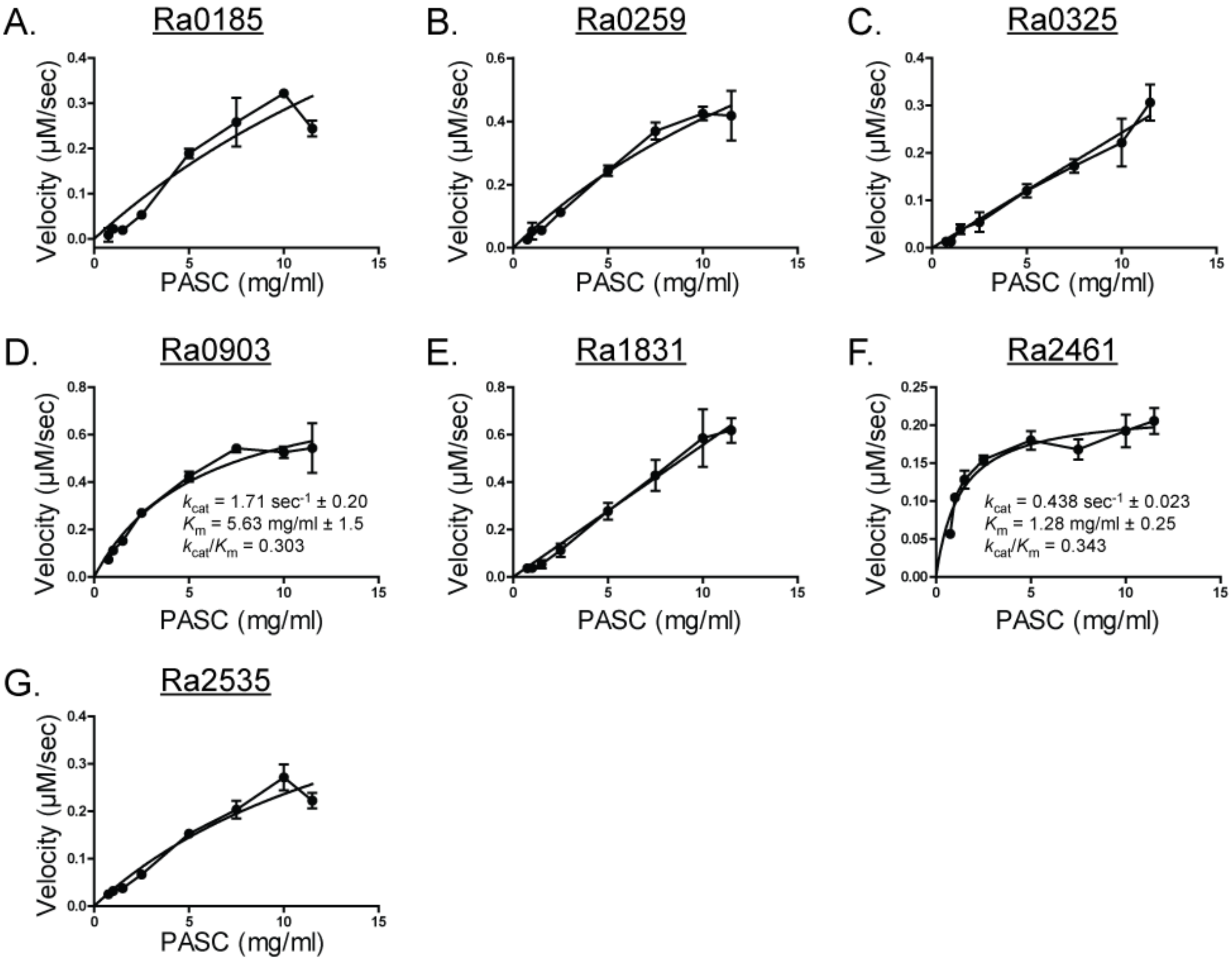

Figure S3. **Enzyme kinetic analysis for individual endoglucanases**. Individual endoglucanases (0.5 μM) were incubated with varying concentrations of PASC. The velocity of each reaction was determined by detecting release of soluble reducing sugars over time by using the PAHBAH reducing sugar assay. Reaction velocities were plotted against PASC concentrations and where applicable fitted to the Michaelis-Menten equation. A, B, C, D, E, F, and G represent Ra0185, Ra0259, Ra0325, Ra0903, Ra1831, Ra2461, and Ra2535, respectively.

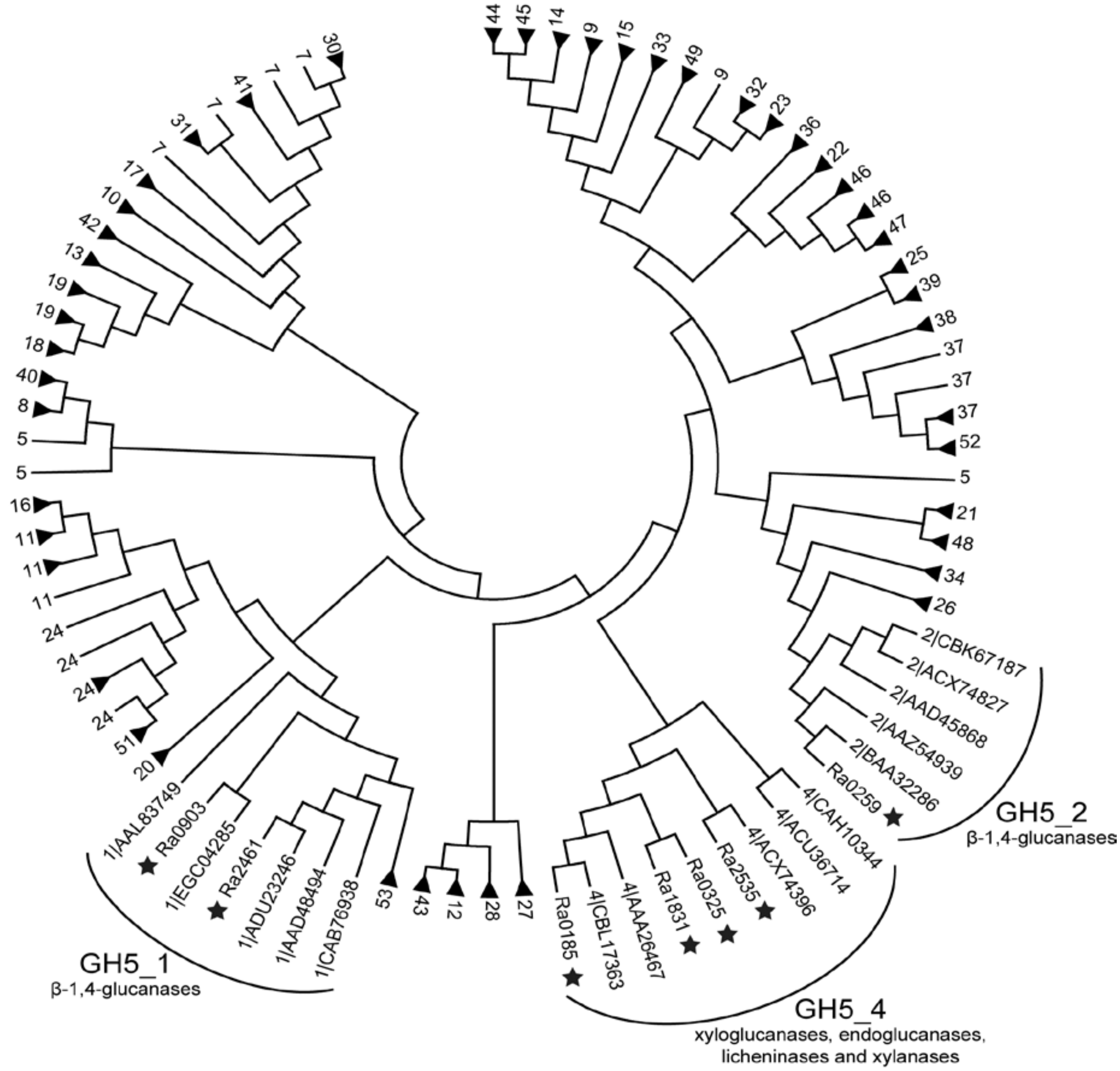

Figure S4. **Phylogenetic analysis of GH5 catalytic domains of *R. albus* 8 endoglucanases.** Representative GH5 protein sequences from each GH5 subfamiliy, as identified by **Aspeborg et al. (32)** (excluding GH5_29, GH5_35, GH5_50 due to low number of representatives) were retrieved from the NCBI (http:www.ncbi.nlm.nih.gov) and trimmed to contain only the catalytic domain by using dbCAN (csbl.bmb.uga.edu/dbCAN/annotate.php) and Pfam (pfam.sanger.ac.uk/). Protein sequences were aligned using ClustalW. The circular cladogram phylogenetic tree was built using MEGA6 (www.megasoftware.net/).

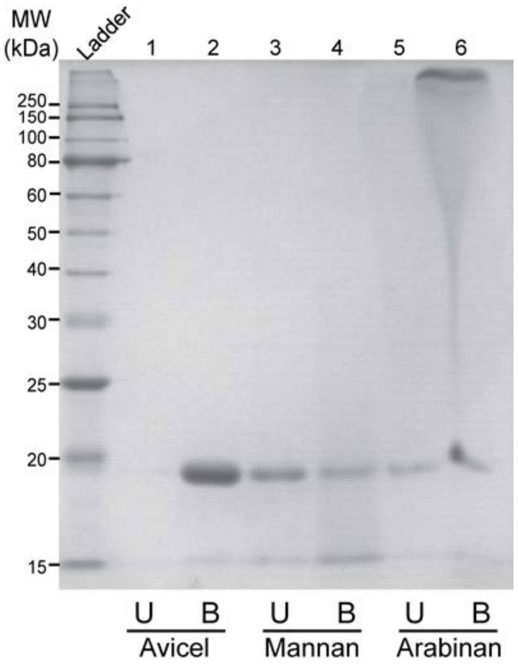

Figure S5. **Substrate binding characteristics of the CBM65 from endoglucanase Ra2535 to insoluble substrate.** Purified Ra2535 TM3 (0.5 mg/ml) was incubated with Avicel, β-1,4 mannan, or debranched arabinan (10 mg/ml) at 4°C for one hour. The unbound fraction or protein remaining in the supernatant was separated from the bound fraction (B) as described in the text. An SDS-PAGE analysis was carried out to visualize (B) and (U) proteins to insoluble substrates.

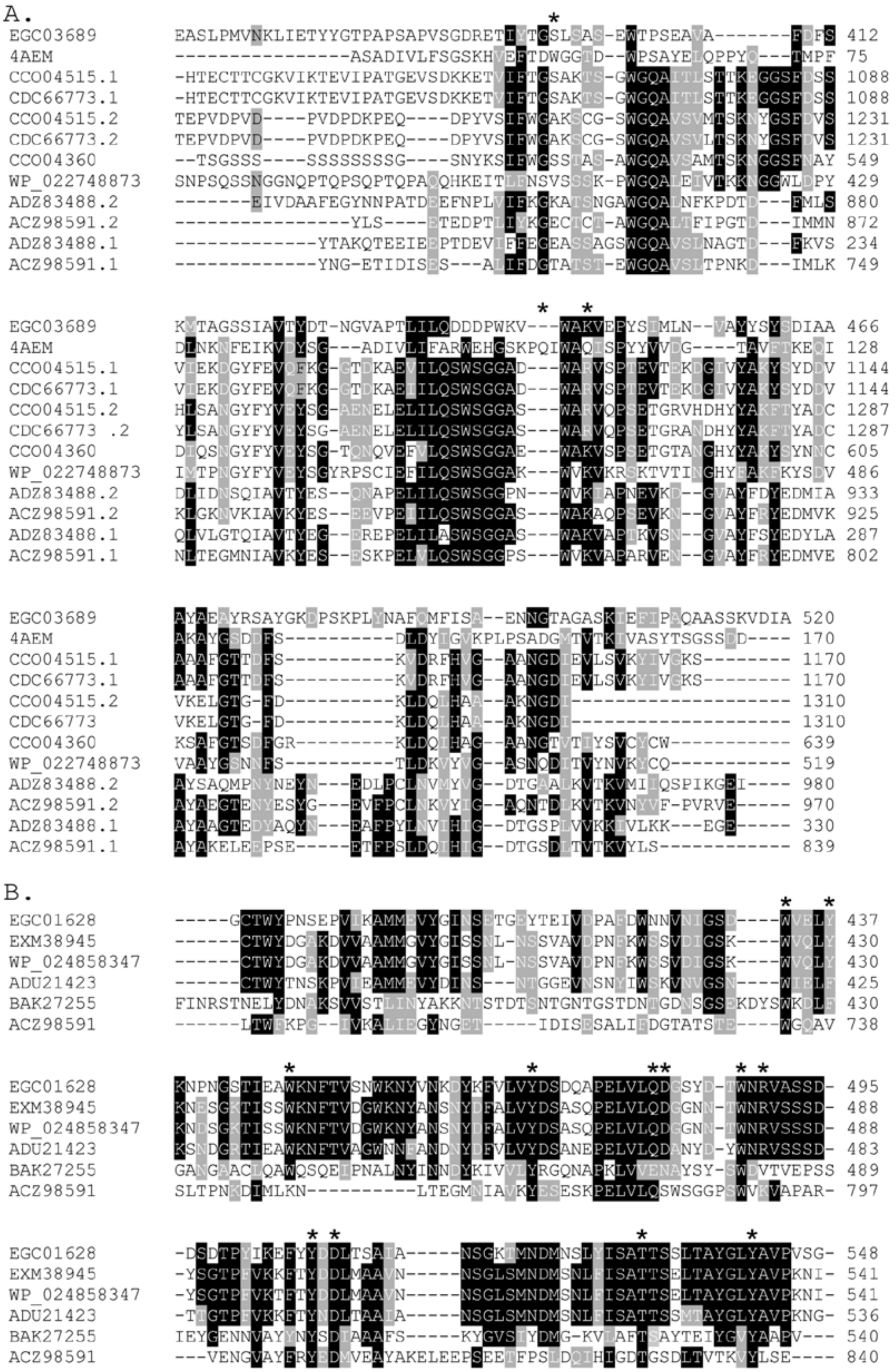
A.
EGC03689 EASLPMVNKLIETYYGTPAPSAPVSGDRETIYTGSLSAS-EWTPSEAVA-------FDFS 412
4AEM ----------------ASADIVLFSGSKHVEFTDWGGID--WPSAYELQPPYQ---TMPF 75
CCO04515.1 -HTECTTCGKVIKTEVIPATGEVSDKKETVIFTGSAKTS-GWGQAITISTTKEGGSFDSS 1088
CDC66773.1 -HTECTTCGKVIKTEVIPATGEVSDKKETVIFTGSAKTS-GWGQAITISTTKEGGSFDSS 1088
CCO04515.2 TEPVDPVD----PVDPDKPEQ----DPYVSIFWGAKSCG-SWGQAVSVMTSKNYGSFDVS 1231
CDC66773.2 TEPVDPVD----PVDPDKPEQ----DPYVSIFWGAKSCG-SWGQAVSVLTSKNYGSFDVS 1231
CCO04360 --TSGSSS----SSSSSSSSG----SNYKSIFWGSSIAS-AWGQAVSAMTSKNGGSFNAY 549
WP_022748873 SNPSQSSNGGNQPTQPSQPTQPAQQHKEITLLNSVSSSK-PWGQALEIVTKKNGGWLDPY 429
ADZ83488.2 -------EIVDAAFEGYNNPATDEEFNPLVIFKGKATSNGAWGQALNFKPDTD---FMLS 880
ACZ98591.2 ----------------YLS----ETEDPTLIYKGECICT-AWGQALTFIPGTD---IMMN 872
ADZ83488.1 -------------YTAKQTEEIEEPTDEVIFFEGEASSAGSWGQAVSINAGTD---FKVS 234
ACZ98591.1 -------------YNG-ETIDISES---ALIFDGTATST-EWGQAVSITPNKD---IMLK 749
EGC03689 KYTAGSSIAVTYDT-NGVAPTLILQDDDPWKV---WAKVEPYSIMLN--VAYYSYSDIAA 466
4AEM DLNKNFEIKVDYSG---ADIVLIFARWEHGSKPQIWAQISPYYVVDG----TAVFTKEQI 128
CCO04515.1 VIEKDGYFEVQFKG-GTDKAEVILQSWSGGAD---WARVSPTEVTEKDGIVYAKYSYDDV 1144
CDC66773.1 VIEKDGYFEVQFKG-GTDKAEIILQSWSGGAD---WARVSPTEVTEKDGIVYAKYSYDDV 1144
CCO04515.2 HLSANGYFYVEYSG-AENELELILQSWSGGAS---WARVQPSETGRVHDHYYAKFTYADC 1287
CDC66773 .2 YLSANGYFYVEYSG-AENELELILQSWSGGAS---WARVQPSETGRANDHYYAKFTYADC 1287
CCO04360 DIQSNGYFYVEYSG-TQNQVEFVLQSWSGGAE---WAKVSPSETGTANGHYYAKYSYNNC 605
WP_022748873 IVTPNGYFYVEYSGYRPSCIEFILQSWSGGAK---WVKVKRSKTVTINGHYEAKEKYSDV 486
ADZ83488.2 DLIDNSQIAVTYES--QNAPELILQSWSGGPN---WVKIAPNEVKD--GVAYFDYEDMIA 933
ACZ98591.2 KLGKNVKIAVKYES--EEVPEIILQSWSGGAS---WAKAQPSEVKN--GVAYFRYEDMVK 925
ADZ83488.1 QLVLGTQIAVTYEG--PREPELILASWSGGAS---WAKVAPTKVSN--GVAYFSYEDYLA 287
ACZ98591.1 NLTEGMNIAVKYES--ESKPELVLQSWSGGPS---WVKVAPARVEN--GVAYFRYEDMVE 802
EGC03689 AYAEAYRSAYGKDPSKPLYNAFQMFISA--ENNGTAGASKIEFIPAQAASSKVDIA 520
4AEM AKAYGSDDFS----------DLDYIGVKPLPSADGMTVTKIVASYTSGSSDD---- 170
CCO04515.1 AAAFGTTDFS----------KVDRFHVG--AANGDIEVLSVKYIVGKS-------- 1170
CDC66773.1 AAAFGTTDFS----------KVDRFHVG--AANGDIEVLSVKYIVGKS-------- 1170
CCO04515.2 VKELGTG-FD----------KLDQLHAA--AKNGDI-------------------- 1310
CDC66773 VKELGTG-FD----------KLDQLHAA--AKNGDI-------------------- 1310
CCO04360 KSAFGTSDFGR---------KLDQIHAG--AANGTVTIYSVCYCW----------- 639
WP_022748873 VAAYGSNNFS----------TLDKVYVG--ASNQDITVYNVKYCQ----------- 519
ADZ83488.2 AYSAQMPNYNEYN---EDLPCLNVMYVG--DTGAALKVTKVMIIQSPIKGEI---- 980
ACZ98591.2 AYAEGTENYESYG---EVFPCLNKVYIG--AQNTDLKVTKVNYVF-PVRVE----- 970
ADZ83488.1 AYAAGTEDYAQYN---EAFPYLNVIHIG--DTGSPLVVKKIVLKK---EGE----- 330
ACZ98591.1 AYAKELEEPSE-----ETFPSLDQIHIG--DTGSDLTVTKVYLS------------ 839
B.
EGC01628 -----GCTWYPNSEPVIKAMMEVYGINSETGEYTEIVDPAEDWNNVNIGSD----WVELY 437
EXM38945 ------CTWYDGAKDVVAAMMGVYGISSNL-NSSVAVDPNFKWSSVDIGSK----WVQLY 430
WP_024858347 ------CTWYDGAKDVVAAMMGVYGISSNL-NSSVAVDPNFKWSSVDIGSK----WVQLY 430
ADU21423 ------CTWYTNSKPVIEAMMEVYDINS---NTGGEVNSNYIWSKVNVGSN----WIELF 425
BAK27255 FINRSTNELYDNAKSVVSTLINYAKKNTSTDTSNTGNTGSTDNTGDNSGSGSKDYSWKDLF 430
ACZ98591 ------LTWFKPG--IVKALIEGYNGET-----IDISESALIFDGTATSTE----WGQAV 738
EGC01628 KNPNGSTIEAWKNFTVSNWKNYVNKDYKFVLVYDSDQAPELVLQDGSYD-IWNRVASSD- 495
EXM38945 KNESGKTISSWKNFTVDGWKNYANSNYDFALVYDSASQPELVLQDGGNN-IWNRVSSSD- 488
WP_024858347 KNDSGKTISSWKNFTVDGWKNYANSNYDFALVYDSASQPELVLQDGGNN-IWNRVSSSD- 488
ADU21423 KSNDGRTIEAWKNFTVAGWNNEANDNYDFVLVYDSANEPELVLQDANYD-YWNRVSSSD- 483
BAK27255 GANGAACLQAWQSQEIPNALNYINNDYKIVVIYRGQNAPKLVVENAYSY-SWDVTVEPSS 489
ACZ98591 SLTPNKDIMLKN----------LTEGMNIAVKYESESKPELVLQSWSGGPSWVKVAPAR- 797
EGC01628 -DSDTPYIKEFYYDDLTSAIA-----NSGKTMNDMNSLYISATTSSLTAYGLYAVPVSG- 548
EXM38945 -YSGTPFVKKFTYDDLMAAVN-----NSGLSMNDMSNLFISATTSELTAYGLYAVPKNI- 541
WP_024858347 -YSGTPFVKKTFTYDDLMAAVN-----NSGLSMNDMSNLFISATTSELTAYGLYAVPKNI- 541
ADU21423 -TTGTPFVKKFTYNDLTAAIA-----NSGLSMNDMSNLFISATTSSMTAYGLYAVPKNG- 536
BAK27255 IEYGENNVAYYNYSDIAAAFS-----KYGVSIYDMG-KVIAFTSAYTEIYGVYAAPV--- 540
ACZ98591 ---VENGVAYFRYEDMVEAYAKELEEPSEETFPSLDQIHIGDTGSDLTVTKVYLSE---- 840

Figure S6. **Amino acid sequence alignments of the analyzed CBMs in Ra2535 and Ra1831 with homologous sequences in the NCBI.** (A) The *R. albus* 8 CBM65 (ECG03689) aligned with its homologous sequences 4AEM, CCO04515, CDC66773, CCO04360, WP_022748873, ADZ83488, and ACZ98591 from *Eubacterium cellulosolvens*, *Ruminococcus* sp. 80/3, *Ruminococcus* sp. CAG:57, *Ruminococcus* sp. 80/3, *Lachnobacterium bovis*, *Clostridium lentocellum* DSM 5427, *Cellulosilyticum ruminicola*, respectively. Asterisk denotes residues shown to be essential for EcCBM65 but are not conserved in the *R. albus* 8 CBM65. Conserved and similar amino acids are shaded in black and gray, respectively. (B) Homologs of the Ra1831 CBM were identified through a search of the NCBI database and aligned using ClustalW. Identical amino acids are highlighted in black and similar amino acids are highlighted in gray. Accession numbers of EGC01628, EXM38945, WP_024858347, ADU21423, BAK27255, and ACZ98591 correspond to sequences from *R. albus* 8, *R. albus* SY3, *R. albus*, *R. albus* 7, *Streptococcus gallolyticus*, and *Cellulosyliticum ruminicola*, respectively. Asterisk denotes residues selected for site-directed mutagenesis of Ra1831 TM2.

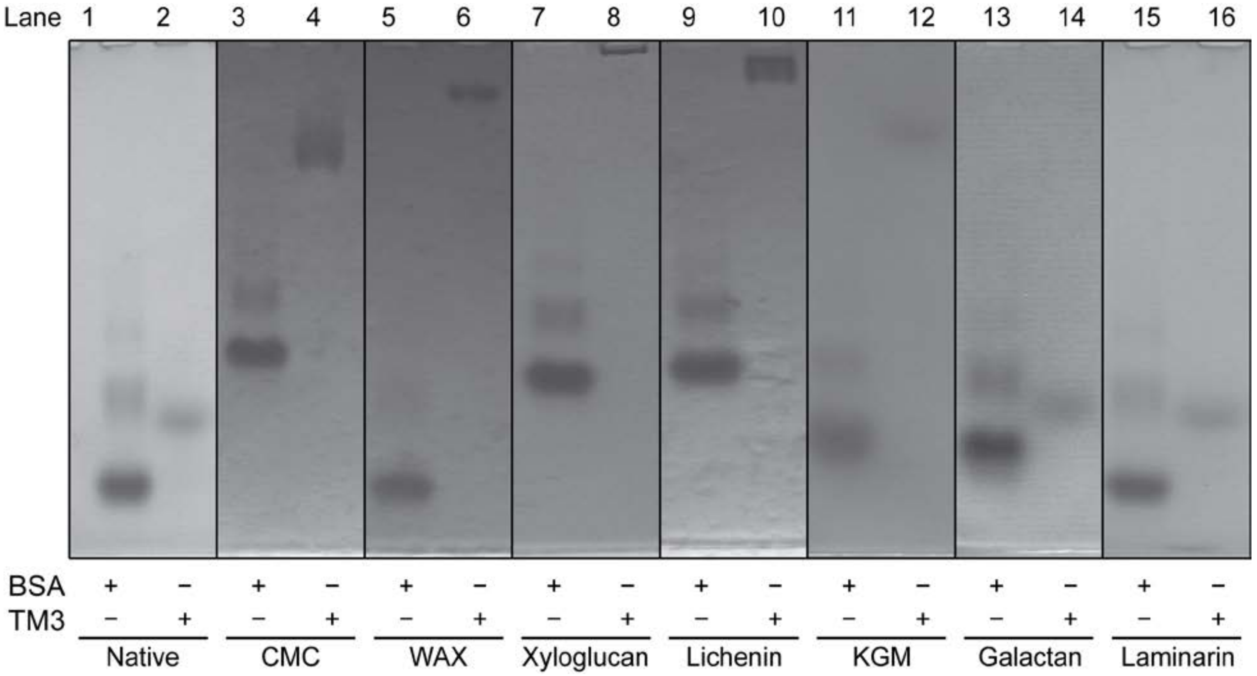

Figure S7. **Substrate binding characteristics of the CBM65 from endoglucanase Ra2535 to soluble substrates.** Native affinity gel electrophoreses were performed to qualitatively analyze the binding of Ra2535 TM3 to soluble polysaccharides (Lane 2, 4, 6, 8, 10, 12, 14, and 16). Migration through a native gel without polysaccharide (Native, Lane 1 and 2) was compared to migration through native gels containing carboxymethyl-cellulose (CMC, Lane 3 and 4), wheat arabinoxylan (WAX, Lane 5 and 6), tamarind xyloglucan (Lane 7 and 8), lichenin (Lane 9 and 10), konjac glucomannan (KGM, Lane 11 and 12), galactan (Lane 13 and 14), and laminarin (Lane 15 and 16). BSA (Lane 1, 3, 5, 7, 9, 11, 13, and 15) was used as a standard for comparison.
